# Short-term superiority in proteinuria control with exploratory longitudinal trajectories: finerenone-dapagliflozin combination in non-diabetic kidney disease

**DOI:** 10.3389/fphar.2026.1918911

**Published:** 2026-10-13

**Authors:** Mali Zhu, Yingyi Deng, Lijun Su, Haixia Zhang, Yun Yang, Lulu Wang

**Affiliations:** 1 Department of Pharmacy, Nanjing Drum Tower Hospital, Clinical College of Nanjing University of Chinese Medicine, Nanjing, China; 2 Department of Pharmacy, Nanjing Drum Tower Hospital, School of Basic Medicine and Clinical Pharmacy, China Pharmaceutical University, Nanjing, China; 3 Department of Pharmacy, Nanjing Drum Tower Hospital, Affiliated Hospital of Medical School, Nanjing University, Nanjing, China; 4 Department of Stomatology, Nanjing Drum Tower Hospital, Affiliated Hospital of Medical School, Nanjing University, Nanjing, China; 5 Hunan Provincial Key Laboratory of the Research and Development of Novel Pharmaceutical Preparations, Changsha Medical University, Changsha, China

**Keywords:** combination therapy, dapagliflozin, finerenone, non-diabetic kidney disease (NDKD), propensity score matching (PSM)

## Abstract

**Introduction:**

Finerenone and dapagliflozin have demonstrated renal protective effects in diabetic kidney disease, but real-world evidence on their combination in non-diabetic kidney disease (NDKD) remains scarce. We assessed whether their combination improves short-term proteinuria control versus monotherapy, with long-term trajectories explored as hypothesis-generating outcomes.

**Methods:**

This retrospective cohort study included 327 NDKD patients admitted to Nanjing Drum Tower Hospital (January 2022–March 2026). Patients were divided into three groups: finerenone monotherapy, dapagliflozin monotherapy, or combination therapy. Propensity score matching (PSM) was used to balance baseline characteristics. Short-term (3 months) efficacy, safety, and exploratory cost analysis were compared. Longitudinal data were analyzed using generalized estimating equations (GEE), with the primary inferential window at 3–6 months and a sensitivity analysis at 3–24 months. Multivariate logistic regression identified predictors of treatment failure in the full unmatched cohort. Exploratory subgroup analyses were performed in membranous nephropathy and hypertension.

**Results:**

After PSM, at 3 months, combination therapy achieved a higher overall remission rate than dapagliflozin monotherapy (69.57% vs. 39.13%, OR = 3.56, 95% CI: 1.50–8.40, *P* = 0.003), but not versus finerenone monotherapy (68.29% vs. 51.22%, OR = 2.05, 95% CI: 0.84–5.05, *P* = 0.115). In the primary 3–6 months GEE analysis, time effects were significant for clinical remission rates and proteinuria; Sensitivity GEE analysis (3–24 months) was consistent. Descriptive analyses from 3 to 24 months showed numerical improvement trends across groups, but these are exploratory. Adverse event rates were low, but the analysis was underpowered to exclude increased hyperkalemia risk. In exploratory cost analysis, the ICER was 22.99 RMB per 1% increase in remission rate versus finerenone and 20.79 RMB versus dapagliflozin; probabilistic sensitivity analysis was broadly consistent with the base case, without judgment of economic superiority. Dapagliflozin monotherapy was independently associated with treatment failure (OR = 2.163, 95% CI: 1.073–4.362, *P* = 0.031) in the full unmatched cohort. Exploratory subgroup analyses suggested potential benefit in membranous nephropathy and hypertension.

**Conclusion:**

Finerenone combined with dapagliflozin provides higher observed short-term (3 months) efficacy in NDKD, with low observed adverse event rates. Long-term outcomes require validation in larger prospective studies.

## 1 Introduction

Chronic kidney disease (CKD), characterized by a reduced estimated glomerular filtration rate (eGFR), increased urinary albumin excretion, or both, has emerged as one of the major public health threats worldwide (Stompór et al., 2023). According to the latest estimates from the Global Burden of Disease Study (GBD, 2021), there were approximately 674 million prevalent cases of CKD worldwide in 2021, accounting for approximately 1.528 million deaths and 44.54 million disability-adjusted life years (DALYs). The corresponding case-fatality rate was 18.50%. Moreover, the disease burden continues to rise (Li et al., 2025). Data from China show that the prevalence of CKD among adults is 10.8%, corresponding to approximately 120 million individuals across all age groups affected by the disease (Luo et al., 2025). Diabetic kidney disease (DKD) is the most common cause of kidney failure; however, a substantial proportion (50%–80%) of patients with CKD have non-diabetic etiologies (Heerspink et al., 2025). Non-diabetic kidney disease (NDKD) refers to a broad category of kidney diseases that excludes DKD, characterized by heterogeneous etiologies and diverse pathological types. According to the KDIGO Clinical Practice Guidelines for Glomerular Diseases and the latest classification criteria, NDKD primarily encompasses primary glomerular diseases, renal involvement in secondary or systemic diseases, and hereditary or congenital kidney diseases (KDIGO, 2024).

Albuminuria is an independent risk factor for the development and progression of CKD; the core treatment goals for NDKD are to sustainably reduce albuminuria levels and slow the decline in eGFR (KDIGO, 2024). Overexpression of the mineralocorticoid receptor (MR) drives renal inflammation, fibrosis, and podocyte injury, thereby exacerbating proteinuria and renal structural remodeling, and represents a key pathological mechanism underlying the progression of CKD (Kim et al., 2023; Ismahel and Docherty, 2025). Finerenone, a third-generation nonsteroidal mineralocorticoid receptor antagonist (nsMRA), exhibits greater selectivity for the MR and exerts potent anti-inflammatory and antifibrotic effects at lower doses. Furthermore, finerenone shows nearly equal distribution between cardiac and renal tissues, thereby significantly reducing the risk of hyperkalemia commonly associated with traditional steroidal mineralocorticoid receptor antagonists (MRAs) (Zhai et al., 2024). The FIDELIO-DKD and FIGARO-DKD trials have demonstrated that finerenone significantly reduces albuminuria, composite renal endpoints, and the risk of cardiovascular events in patients with type 2 diabetes mellitus (T2DM) and CKD (Bakris et al., 2020; Pitt et al., 2021). However, the patient populations in the aforementioned pivotal trials consisted exclusively of individuals with DKD, and evidence regarding the efficacy and safety of finerenone in patients with NDKD remains very limited. To date, real-world evidence on finerenone in the NDKD population is scarce, with only a few studies having examined its efficacy and safety. Therefore, the clinical value of finerenone in patients with NDKD urgently requires validation through real-world studies (Albakr et al., 2025). Sodium-glucose cotransporter 2 inhibitors (SGLT2i) represent another class of agents that has achieved breakthrough progress in the treatment of CKD in recent years. These agents have been shown to slow the progression of DKD and reduce the risk of cardiovascular events and mortality in affected patients (Tesař, 2022). The DAPA-CKD trial demonstrated that dapagliflozin significantly reduced the risk of a composite renal endpoint and all-cause mortality in patients with CKD, with or without T2DM (Heerspink et al., 2020). Pre-specified subgroup analyses of this trial further confirmed that the renal protective effect of dapagliflozin in patients with NDKD is consistent with that observed in patients with DKD (Jongs et al., 2021). Additionally, the EMPA-KIDNEY trial further confirmed that SGLT2i confer benefits to a broader spectrum of patients with CKD (EMPA-KIDNEY Collaborative Group, 2023). The KDIGO guidelines currently recommend SGLT2i inhibitors as first-line standard-of-care therapy for CKD (KDIGO, 2024).

Finerenone primarily targets and blocks the inflammatory and fibrotic pathways mediated by excessive MR activation, whereas dapagliflozin exerts its effects through multiple mechanisms, including reducing glomerular hyperfiltration and improving renal oxygenation and metabolic homeostasis. Theoretically, the combination of these two agents may produce a synergistic effect in reducing proteinuria and slowing the decline in renal function (Asirvatham et al., 2024). Currently, most available evidence on the combination of finerenone and dapagliflozin comes from studies in DKD. In contrast, for the NDKD population—which is characterized by greater etiological heterogeneity and more complex pathophysiological mechanisms—the short-, medium-, and long-term clinical efficacy, safety, and cost-effectiveness of this combination remain unclear. Notably, there is a particular lack of high-quality evidence derived from real-world data in the Chinese population. So, we hypothesized that finerenone combined with dapagliflozin would achieve superior short-term proteinuria control compared with either monotherapy; long-term renal trajectories were explored as hypothesis-generating outcomes.

## 2 Materials and methods

### 2.1 Study design and data source

A retrospective cohort study was conducted in patients with NDKD who were admitted to Nanjing Drum Tower Hospital, between January 2022 and March 2026 (with finerenone-containing regimens available only after its NMPA approval in June 2022; patients enrolled before June 2022 received dapagliflozin monotherapy). Index date was defined as the date of first prescription of either finerenone or dapagliflozin. Combination-therapy group strictly enrolled patients with simultaneous initiation of both finerenone and dapagliflozin; patients receiving sequential add-on therapy (one agent initiated first, the second added subsequently) were excluded from this study. Patients who discontinued or switched assigned regimen within the first 30 days after index date were excluded at baseline. Participants who switched treatment regimen after the 30-day exposure window were censored at the date of regimen switch in longitudinal analyses.

Inclusion criteria: (1) Age ≥18 years, meeting at least one of the following conditions: (a) eGFR <60 mL/min/1.73 m^2^, calculated using the CKD-EPI 2009 multi-ethnic equation. According to local drug labelling, eGFR ≥25 mL/min/1.73 m^2^ was required for both finerenone-containing regimens and dapagliflozin monotherapy. (b) Urinary albumin-to-creatinine ratio ≥30 mg/g, or 24-h urinary albumin excretion ≥30 mg/24 h, or urinary protein ≥1 + accompanied by confirmatory quantitative albumin/protein testing to confirm persistent proteinuria; (c) Diagnosis of CKD fulfilling KDIGO 2024 criteria (persistent kidney injury or decreased eGFR for ≥3 months), documented in outpatient or inpatient medical records. (2) Regular use of finerenone and/or dapagliflozin for >1 month (3) Absence of a diagnosis of diabetic kidney disease.

Exclusion criteria: (1) Pregnancy or lactation; (2) History of chemotherapy or treatment with alkylating agents for malignant tumors within the preceding 2 years; (3) History of dialysis for >1 month and/or history of solid organ or bone marrow transplantation; (4) History of immunosuppressive therapy for primary kidney disease or systemic diseases within the preceding 6 months; (5) History of hereditary kidney diseases, such as polycystic kidney disease; (6) Incomplete clinical records and follow-up data.

The study flowchart is shown in Figure 1. Data were extracted from the Hospital Information System (HIS). A unique identification code was generated using the names of study participants, outpatient/inpatient ID numbers, ages, and genders. This unique identification code was then used to match data from various forms and to link the follow-up records of participants. Baseline patient data were collected, including demographic information [age, sex, weight, body mass index (BMI)], pathological type [membranous nephropathy (MN), IgA nephropathy, primary glomerular disease, secondary/other nephropathies], comorbidities (hypertension, cardiovascular complications, hyperlipidemia), and concomitant medications (angiotensin converting enzyme inhibitors, angiotensin II receptor blockers, calcium channel blockers, diuretics, beta-blockers, alpha-blockers, central-acting antihypertensives), etc. Cross-tabulation was performed for data including patient demographics, medication regimens, laboratory test results, and treatment costs. All patients received standard therapy. They were divided into finerenone group, dapagliflozin group and combination therapy group according to their treatment regimens. Patients in finerenone group received finerenone initiated at 10–20 mg/day based on eGFR and serum potassium, and titrated within 4 weeks according to serum potassium, dose reduction or temporary discontinuation was implemented for serum potassium >5.5 mmol/L; patients in dapagliflozin group received oral dapagliflozin (10 mg/day), and patients in the combination therapy group received combination therapy with finerenone (10–20 mg/day) combined with dapagliflozin (10 mg/day). Standard background therapy consisted of guideline-directed RAAS inhibitors, antihypertensive medications, lipid-lowering agents and supportive nephroprotective measures, with the specific agents and doses selected according to each patient’s individual clinical needs. Serum potassium and renal function were routinely monitored according to local clinical practice. Relevant indicators were collected at 3, 6, 9, 12, 15, 18, 21, and 24 months, and longitudinal analysis was performed combining data from all three groups.

**FIGURE 1:**
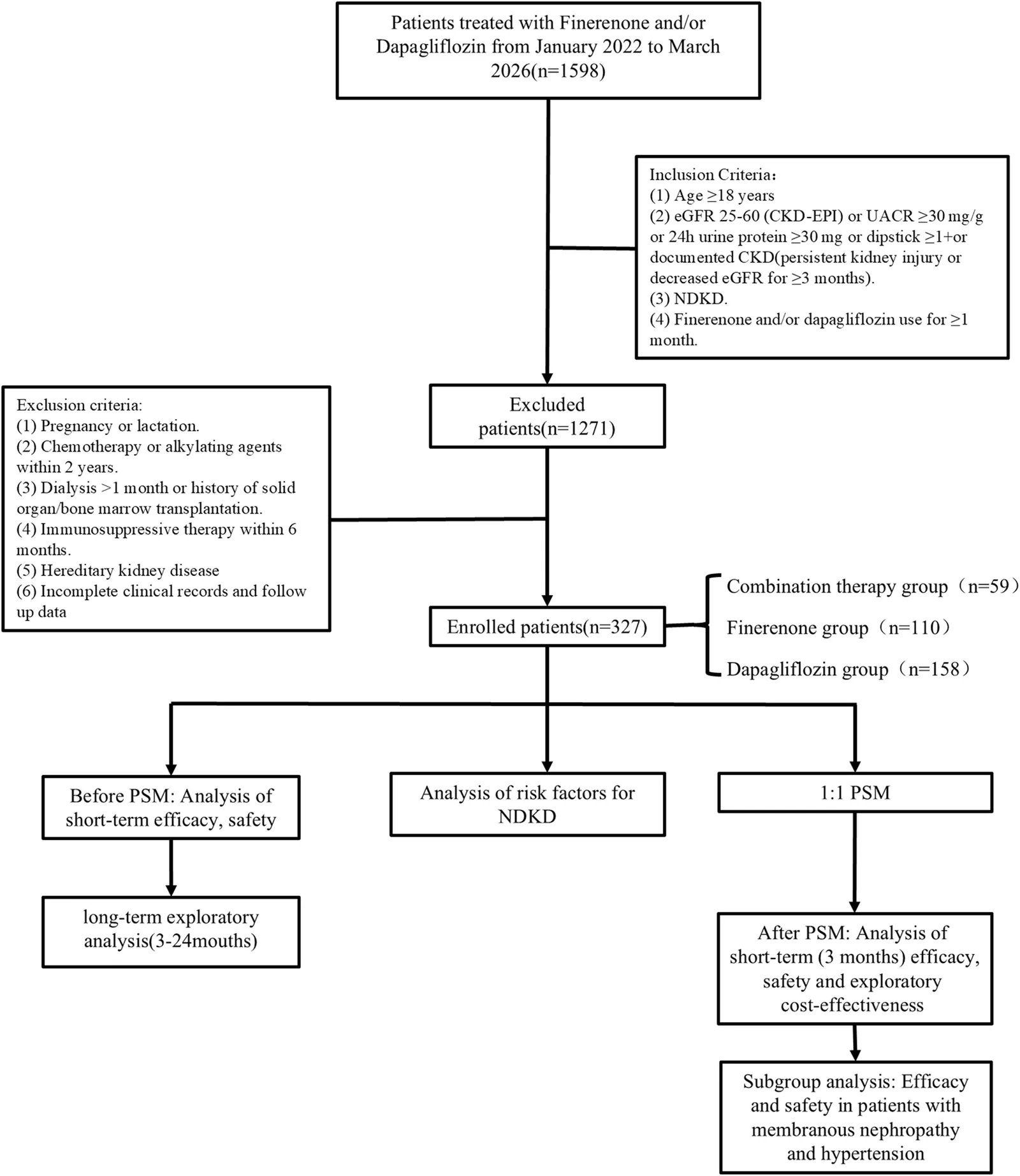
Flow chart of the study. CKD, chronic kidney disease; PSM, propensity score matching; NDKD, non-diabetic kidney disease.

This retrospective anonymized study was approved by the Ethics Committee of Nanjing Drum Tower Hospital (Approval No. 2025-1152-01). Written informed consent was waived by the ethics committee due to retrospective de-identified data analysis. The study complied with the 1964 Declaration of Helsinki and its subsequent amendments.

### 2.2 Outcome measures

#### 2.2.1 Clinical efficacy

Because there is no universally accepted remission definition for NDKD, we used a pre-specified, exploratory definition with reference to the KDIGO guidelines (KDIGO, 2024), the criteria established for this study are as follows:

Complete remission: 24-h urinary protein < 0.3 g/24 h or urine albumin-to-creatinine ratio (UACR) < 30 mg/g, serum albumin ≥35 g/L, and stable renal function (eGFR decline <15% and no renal replacement therapy); Partial remission: 24-h urinary protein < 3.5 g/24 h and a decrease of at least 50% from baseline or UACR <300 mg/g and a decrease of at least 50% from baseline, together with normal (≥35 g/L) or improved (an increase from baseline of ≥3 g/L) plasma albumin levels and stable renal function (eGFR decline < 15% and no renal replacement therapy); No response: Failure to meet any of the above criteria. Patients were classified hierarchically: complete remission took precedence; patients meeting complete remission criteria were not counted as partial remission. Overall remission rate = (Number of patients with complete remission + Number with partial remission)/Total number of patients × 100%.

With reference to the KDIGO guidelines (KDIGO, 2024), a sensitivity analysis was performed using remission criteria commonly applied to IMN (Idiopathic Membranous Nephropathy) in the subgroup of patients with available 24-h urinary protein measurements at both baseline and 3 months. The KDIGO 2024 definition about IMN was applied as follows: complete remission was defined as 24-h urinary protein < 0.3 g/24 h, serum albumin ≥ 35 g/L, and stable renal function (eGFR decline <15% and no renal replacement therapy); partial remission was defined as 24-h urinary protein < 3.5 g/24 h with a decrease of ≥50% from baseline, together with normal (≥35 g/L) or improved (an increase from baseline of ≥3 g/L) plasma albumin levels and stable renal function (eGFR decline < 15% and no renal replacement therapy). No response: Failure to meet any of the above criteria. Because 24-h urinary protein quantification was not available for all patients, this analysis was restricted to the subgroup with complete 24-h urinary protein data and was considered exploratory.

#### 2.2.2 Efficacy

The primary efficacy endpoints: 24-h urine volume, 24-h urinary protein, UACR, eGFR, and serum creatinine (Scr). Additionally, the following derived measures were assessed: percentage reductions in 24-h urinary protein and UACR, as well as rates of change in eGFR, Scr, systolic blood pressure (SBP), and diastolic blood pressure (DBP). eGFR was calculated using the CKD-EPI equation. Urinary albumin and creatinine concentrations in morning urine specimens were measured using an immunoturbidimetric assay, from which the UACR was derived. Scr levels were measured using a biochemical analyzer.

Secondary efficacy endpoints: SBP, DBP, c-reactive protein (CRP), hemoglobin (Hb), platelet count, serum albumin, alkaline phosphatase, uric acid, serum phosphorus, serum potassium, serum sodium, serum chloride, serum bicarbonate, triglycerides, total cholesterol, high-density lipoprotein cholesterol (HDL-C), and low-density lipoprotein cholesterol (LDL-C).

#### 2.2.3 Safety

The primary safety endpoint was the incidence of adverse events, adverse reactions included hyperkalemia, genitourinary tract infections, abdominal distension, dizziness, diarrhea, nausea, and hypotension.

#### 2.2.4 Economic indicators

The total cost of drug treatment includes direct costs, indirect costs, and hidden costs. Given the lack of uniform quantification standards and accounting methods for indirect and hidden costs in real-world settings, this study conducted an economic analysis based solely on direct medical costs from a health system perspective (Xu et al., 2023). According to the 2022 pricing standards of China’s National Medical Insurance Drug List, dapagliflozin (10 mg) is priced at 4.36 RMB, and finerenone (10 mg) is priced at 7.03 RMB after its market approval in China in June 2022 and subsequent inclusion in the national medical insurance scheme in 2023. Finerenone cost was calculated at 10 mg/day, the initial and most commonly used dose in this cohort. Because dose titration to 20 mg/day was permitted, this may underestimate the actual drug cost. Owing to patient privacy restrictions, detailed individual-level medical cost data could not be obtained. Therefore, this study employed a simplified cost-effectiveness analysis method. The cost components were defined in accordance with the Chinese Guidelines for Pharmacoeconomic Evaluation and similar studies (Tang et al., 2001; Zhao et al., 2003; Association, 2020), including: drug acquisition costs (costs for a 3-month course of finerenone and/or dapagliflozin); monitoring and testing costs (costs of baseline examinations and 3-month follow-up examinations). The testing items included: a complete blood biochemistry panel, complete blood count, urinalysis with quantitative urine sediment analysis, urine protein screening, and morning urine red blood cell morphology examination.

Economic evaluation was performed using pharmacoeconomic methods. When there was no difference in efficacy and safety between the combination therapy group and the monotherapy groups, a cost-minimization analysis was adopted; when a difference existed, cost-effectiveness analysis was used. The Incremental Cost-Effectiveness ratio (ICER) = (C of combination therapy − C of monotherapy)/(E of combination therapy − E of monotherapy) = ΔC/ΔE. To assess the robustness of the base-case analysis, one-way sensitivity analyses were performed, with each parameter varied within ±20% of its baseline value. In addition, probabilistic sensitivity analysis (PSA) was performed using Monte Carlo simulation with 1,000 iterations. Cost parameters were assigned Gamma distributions (standard deviation set at 20% of the mean), and effect parameters were assigned Beta distributions (α = number of responders, β = number of non-responders). In each iteration, values were randomly sampled simultaneously from all parameter distributions, and the ICER was calculated in RMB per 1% increase in remission rate. Results are presented as median and 95% uncertainty interval (2.5th–97.5th percentiles of 1,000 Monte Carlo simulations), along with a cost-effectiveness plane scatter plot.

### 2.3 Analysis of influencing factors

Univariate and multivariate binary logistic regression analyses were performed to identify risk factors associated with treatment outcomes. Variables with *P* < 0.1 in the univariate analysis were entered into the multivariate logistic regression model using the Enter method. The multivariate analysis was conducted in the full unmatched cohort (n = 327), and adjusted odds ratios (OR) with 95% confidence intervals were reported. Results from this analysis are presented as exploratory risk-factor associations.

### 2.4 Subgroup analysis

Using a stratified analysis approach, patients with MN and those with hypertension were identified from the post-PSM cohort as separate subgroups, and targeted efficacy and safety analyses were performed for each. The primary outcome measures for the subgroup analyses were consistent with those described above. The analyses focused on investigating differences in treatment efficacy among the three regimens within each subgroup, while also evaluating the safety profiles of the different regimens in these populations.

### 2.5 Statistical analysis

Data were analyzed using SPSS 27.0 and RStudio (version 4.5.2). Missing data mainly occurred due to missed follow-up appointments, patient transfer to external hospitals, or incomplete laboratory tests. Missing data were handled as follows: records with more than 20% missing values were excluded, and those with ≤20% missing values were imputed using median imputation. PSM was used to balance baseline confounding factors between groups. A logistic regression model was fitted with all relevant baseline confounding factors as independent variables to derive the propensity score (PS). A 1:1 nearest-neighbor matching method without replacement was employed, with a caliper width set at 0.1 standard deviations (SD). For the matched data, Categorical variables were expressed as frequencies (percentages) and compared between groups using Pearson chi-square test (two-sided) when all expected cell counts were ≥5, or Fisher’s exact test when any expected cell count was <5. Normally distributed continuous variables were expressed as mean ± standard deviation ( $\bar{x}\pm s$ ) and compared using the independent-samples t-test. Non-normally distributed continuous variables were expressed as median [M (Q1, Q3)] and compared using the Mann–Whitney U test. *P < 0.05, **P < 0.01, **P < 0.001. Longitudinal outcomes (clinical remission, 24-h urinary protein, its rate of reduction and eGFR) were analysed using generalized estimating equations (GEE) with an exchangeable working correlation structure and robust sandwich standard errors; models included time, treatment group and their interaction as fixed effects. The primary model was restricted to the 3∼6-month window, and a sensitivity model included all time points (3–24 months) with per-time-point denominators in the full unmatched cohort.

## 3 Results

### 3.1 Patient characteristics

This study included a total of 327 subjects. The mean baseline eGFR in the overall cohort was 87.85 ± 31.56 mL/min/1.73 m^2^, reflecting the inclusion criteria permitting entry via proteinuria regardless of eGFR level; only a minority of patients (n = 56, 17.13%) were enrolled through the low-eGFR criterion. The combination therapy group (n = 59) consisted of 39 men and 20 women, with a median age of 47.00 (36.00, 66.00) years. Finerenone group (n = 110) included 59 men and 51 women, with a median age of 50.00 (38.00, 60.25) years. Dapagliflozin group (n = 158) comprised 83 men and 75 women, with a median age of 47.00 (35.75, 57.00) years. PSM was performed on the following clinical baseline variables: age, sex, BMI, SBP, DBP, histological type, hypertension, UACR, 24-h urinary protein, serum albumin, and eGFR. After PSM, baseline characteristics were well balanced between the combination therapy group and each monotherapy group, with no statistically significant differences (Table 1; Supplementary Table S1).

**TABLE 1:** Comparison of baseline characteristics among the three groups after PSM.

Variable	Combination (n = 41)	Finerenone (n = 41)	t/Z/X^2^	*P*	Combination (n = 46)	Dapagliflozin (n = 46)	t/Z/X^2^	*P*
Age, years	50.98 ± 18.52	49.32 ± 16.51	−0.428	0.670	51.63 ± 16.20	49.43 ± 12.96	−0.718	0.475
Sex, male, n (%)	25 (60.98)	28 (68.29)	0.480	0.488	29 (63.04)	26 (56.52)	0.407	0.524
BMI, kg/m^2^	24.53 ± 3.42	24.38 ± 3.47	−0.196	0.845	24.39 (23.13, 27.15)	24.32 (21.72, 25.95)	−0.906	0.365
SBP, mmHg	122.00 (119.00, 137.00)	132.00 (117.50, 138.00)	−0.656	0.512	122.00 (112.75, 127.50)	127.00 (110.00, 139.25)	−1.028	0.304
DBP, mmHg	75.00 (74.00, 85.50)	83.00 (76.00, 86.00)	−1.469	0.142	75.00 (70.75, 79.25)	80.00 (70.75, 88.00)	−1.585	0.113
Histological type	​	​	2.267	0.519	​	​	7.503	0.056
MN, n (%)	21 (51.22)	21 (51.22)	​	​	30 (65.22)	20 (43.48)	​	​
IgA nephropathy, n (%)	6 (14.63)	9 (21.95)	​	​	9 (19.57)	21 (45.65)	​	​
Primary glomerular disease, n (%)	7 (17.07)	3 (7.32)	​	​	4 (8.70)	2 (4.35)	​	​
Secondary/Other nephropathy, n (%)	7 (17.07)	8 (19.51)	​	​	3 (6.52)	3 (6.52)	​	​
Hypertension, n (%)	27 (65.85)	24 (58.54)	0.467	0.494	31 (67.39)	24 (52.17)	2.215	0.137
UACR, mg/g	1174.00 (414.85, 3588.70)	1866.10 (432.70, 3715.00)	−0.478	0.633	1055.05 (297.03, 3676.43)	774.30 (316.50, 3427.88)	−0.297	0.767
24-h urinary protein, mg	2339.00 (1050.50, 4412.00)	2696.00 (920.50, 6037.50)	−0.023	0.982	2278.00 (949.50, 4354.00)	1554.00 (635.75, 4956.50)	−0.223	0.824
Serum albumin, g/L	39.20 (31.25, 41.95)	34.70 (28.20, 42.40)	−0.825	0.409	39.30 (34.85, 42.40)	38.50 (33.95, 42.05)	−0.461	0.645
eGFR, mL·min^−1^·1.73 m^−2^	84.12 ± 32.67	86.86 ± 41.77	0.330	0.742	83.58 ± 28.19	84.97 ± 31.34	0.224	0.823

BMI, body mass index; SBP, systolic blood pressure; DBP, diastolic blood pressure; MN, membranous nephropathy; UACR, urinary albumin-to-creatinine ratio; eGFR, estimated glomerular filtration rate.

### 3.2 Short-term efficacy

Before PSM, the overall clinical remission rate in the combination therapy group was 64.41%, compared with 48.18% in the finerenone group (OR = 1.95, 95% CI: 1.02–3.73, *P* = 0.044) and 46.20% in the dapagliflozin group (OR = 2.11, 95% CI: 1.14–3.91, *P* = 0.017). However, due to baseline imbalances, these results should be interpreted as preliminary (Supplementary Table S2). After PSM, at 3 months of treatment, the overall clinical remission rate in the combination therapy group was 68.29% compared with 51.22% in the finerenone group, with an OR of 2.05 (95% CI: 0.84–5.05; *P* = 0.115). Compared with the dapagliflozin group, the overall remission rate in the combination therapy group was 69.57% versus 39.13%, with an OR of 3.56 (95% CI: 1.50–8.40; *P* = 0.003) (Table 2). In the sensitivity analysis among post-PSM patients with available 24-h urinary protein data at 3 months, the overall clinical remission rate was 70.59% in the combination group versus 52.94% in the finerenone group (OR = 2.132, 95% CI: 0.786–5.780; *P* = 0.134), and 66.67% versus 40.48% in the dapagliflozin group (OR = 2.941, 95% CI: 1.188–7.299; *P* = 0.018) (Supplementary Table S3). The direction of the between-group differences was consistent with the primary analysis: combination therapy was superior to dapagliflozin monotherapy, whereas the difference versus finerenone monotherapy did not reach statistical significance.

**TABLE 2:** Comparison of clinical efficacy at 3 months among the three groups after PSM.

Response criteria	Combination (n = 41)	Finerenone (n = 41)	OR (95% CI)	*P*	Combination (n = 46)	Dapagliflozin (n = 46)	OR (95% CI)	*P*
Overall clinical remission rate (%)	28 (68.29)	21 (51.22)	2.05 (0.84–5.05)	0.115	32 (69.57)	18 (39.13)	3.56 (1.50–8.40)	0.003
Complete remission (%)	10 (24.39)	10 (24.39)	​	​	11 (23.91)	8 (17.39)	​	​
Partial remission (%)	18 (43.90)	11 (26.83)	​	​	21 (45.65)	10 (21.74)	​	​
No response (%)	13 (31.71)	20 (48.78)	​	​	14 (30.43)	28 (60.87)	​	​

OR, odds ratio; CI, confidence interval.

Before PSM, UACR in the combination therapy group was significantly lower than that in finerenone group (*P* < 0.05), and the 24-h urinary protein level was also significantly lower than that in finerenone group (*P* < 0.05). The rate of change in SBP and DBP was lower than that in finerenone group, with statistically significant differences (*P* < 0.05). The rate of reduction in 24-h urinary protein in the combination therapy group was significantly higher than that in dapagliflozin group (*P* < 0.05) (Supplementary Table S4). Details of secondary outcome measures are provided in Supplementary Table S5. After PSM, the rate of reduction in 24-h urinary protein in the combination therapy group was higher than that in finerenone group, with a statistically significant difference (*P* < 0.05), but no significant difference was observed compared with dapagliflozin group (*P* > 0.05). Serum albumin levels in the combination therapy group were higher than those in finerenone group, with a statistically significant difference (*P* < 0.05), but no significant difference was observed compared with dapagliflozin group (*P* > 0.05). Comparisons of other indicators among the three groups showed no statistically significant differences (*P* > 0.05). Additionally, 24-h urinary protein and UACR levels in all three groups decreased significantly after treatment compared with baseline, the combination therapy group showed a greater magnitude of decrease in both indicators, with some comparisons reaching *P* < 0.001. In finerenone group and dapagliflozin group, 24-h urinary protein and UACR also decreased significantly, with some indicators showing statistically significant changes (*P* < 0.01). While in dapagliflozin group, SBP and DBP decreased significantly from baseline (*P* < 0.05) (Table 3). Other secondary outcomes are in Supplementary Table S6.

**TABLE 3:** Comparison of primary efficacy and partial secondary efficacy outcomes at 3 months among the three groups after PSM.

Variable	Combination (n = 41)	Finerenone (n = 41)	t/Z	*P*	Combination (n = 46)	Dapagliflozin (n = 46)	t/Z	*P*
24-h urine volume, ml	1800.00 (1425.00, 2422.50)	1700.00 (1000.00, 2450.00)	−0.823	0.410	2000.00 (1500.00, 2500.00)	2000.00 (1650.00, 2312.50)	−0.100	0.920
UACR, mg/g	293.00 (83.30, 1005.10)***	566.95 (157.00, 1323.38)**	−1.212	0.226	293.00 (90.50, 1030.60)**	352.70 (142.85, 779.55)**	−0.255	0.799
24-h urinary protein, mg	669.50 (307.75, 1020.75)***	890.00 (439.25, 2916.00)*	−1.631	0.103	664.00 (343.00, 1281.00)***	744.50 (360.75, 2004.75)**	−0.477	0.633
eGFR, mL·min^−1^·1.73 m^−2^	84.04 ± 39.20	84.58 ± 38.82	−0.061	0.952	83.68 ± 30.98	82.15 ± 30.36	0.235	0.815
Scr, µmol/L	83.00 (66.00, 117.00)	86.00 (62.00, 116.00)	−0.077	0.939	84.00 (66.00, 117.00)	77.00 (63.50, 115.25)	−0.423	0.672
Δ SBP (%)	−4.50 ± 11.96	−2.77 ± 12.87	−0.377	0.709	−1.64 (−9.84, 7.14)	−2.35 (−19.66, 0.00)	−1.101	0.271
Δ DBP (%)	−4.65 ± 15.89	−8.06 ± 12.38	0.626	0.536	−2.70 (−13.33, 6.67)	−8.49 (−25.77, 0.00)	−1.351	0.177
Δ UACR (%)	65.11 (37.91, 76.59)	60.02 (32.24, 80.71)	−0.520	0.603	60.48 (37.22, 74.65)	54.97 (25.98, 79.38)	−1.021	0.307
Δ 24-h urinary protein (%)	58.94 (46.83, 77.59)	51.20 (23.00, 67.43)	−2.012	0.044	55.75 (45.85, 69.44)	51.20 (34.69, 63.86)	−1.295	0.195
Δ eGFR (%)	0.27 (−6.38, 6.39)	−0.28 (−9.84, 10.16)	−0.218	0.827	−0.80 (−7.06, 4.26)	−2.01 (−13.45, 7.92)	−0.451	0.652
Δ Scr (%)	−1.43 (−7.07, 6.71)	0.00 (−8.73, 9.31)	−0.123	0.902	0.83 (−7.04, 8.93)	1.76 (−7.63, 11.85)	−0.530	0.596
SBP, mmHg	119.68 ± 11.71	126.08 ± 13.43	−1.401	0.172	120.00 (112.00, 124.00)	115.00 (100.00, 130.00)*	−0.636	0.524
DBP, mmHg	74.11 ± 13.79	77.33 ± 13.15	−0.646	0.523	75.00 (68.00, 80.00)	71.00 (62.50, 80.00)**	−1.030	0.303
Serum albumin, g/L	39.86 ± 4.69	36.92 ± 6.96	2.050	0.045	41.00 (36.93, 44.00)	41.60 (35.43, 43.08)	−0.667	0.505

Δ, percent change from baseline; abbreviations as in Table 1. Additional abbreviations: Scr, serum creatinine. *indicates comparison with baseline. Statistical significance is indicated as follows: *P < 0.05, **P < 0.01, ***P < 0.001.

### 3.3 Short-term safety

Before PSM, there were no statistically significant differences in adverse reaction incidence between the combination therapy group and either monotherapy group (*P* > 0.05) (Supplementary Table S7) After PSM, the incidence of adverse reactions was 9.76% in both the combination therapy group and finerenone group, with no statistically significant difference between the two groups (*P* > 0.05). The incidence of adverse reactions was 8.70% in the combination therapy group and 6.52% in dapagliflozin group, again with no statistically significant difference (*P* > 0.05). The reported adverse reactions were primarily hyperkalemia and urinary tract infections, all of which were mild in severity. No serious adverse events occurred (Table 4).

**TABLE 4:** Comparison of safety outcomes at 3 months among the three groups after PSM.

Adverse reaction	Combination (n = 41)	Finerenone (n = 41)	P	Combination (n = 46)	Dapagliflozin (n = 46)	*P*
Incidence of adverse reactions (%)	4 (9.76)	4 (9.76)	1.000	4 (8.70)	3 (6.52)	1.000
Hyperkalemia (%)	4 (9.76)	4 (9.76)	​	4 (8.70)	0 (0.00)	​
Urinary tract infection (%)	0 (0.00)	0 (0.00)	​	0 (0.00)	2 (4.35)	​
Hypoglycemia (%)	0 (0.00)	0 (0.00)	​	0 (0.00)	1 (2.17)	​

### 3.4 Short-term cost-effectiveness

Given the differences in efficacy between the combination therapy group and the two monotherapy groups, a cost-effectiveness analysis was employed to compare economic outcomes. After PSM, the per-patient treatment cost was 1,680.22 RMB in the combination therapy group, 1,287.82 RMB in finerenone group, and 1,047.52 RMB in dapagliflozin group. Drug costs differed among the three treatment regimens, whereas diagnostic costs were the same across all groups (Supplementary Table S8). After 3 months of treatment, the combination therapy showed superior efficacy compared with finerenone monotherapy. Every 1% increase in the clinical remission rate was associated with an additional cost of approximately 22.99 RMB. Compared with dapagliflozin monotherapy, the combination therapy also showed superior efficacy, with an additional cost of approximately 20.79 RMB per 1% increase in the clinical remission rate.

One-way sensitivity analyses (costs and effects varied by ±20%) yielded ICER ranges of 18.39–27.59 RMB/% (cost variation) and 19.15–28.75 RMB/% (effect variation) for combination vs. finerenone, and 16.63–24.94 RMB/% (cost variation) and 17.32–25.97 RMB/% (effect variation) for combination vs. dapagliflozin, indicating robust base-case findings (Table 5).

**TABLE 5:** Cost-effectiveness analysis at 3 months of the combination therapy group and monotherapy group after PSM, with costs and effect varied by ±20%.

Group	C varied by ±20%					E varied by ±20%				
	C (RMB)	E (%)	ΔC (RMB)	ΔE (%)	ICER (RMB/%)	C (RMB)	E (%)	ΔC (RMB)	ΔE (%)	ICER (RMB/%)
Combination	−20% 1344.18	​	313.92	17.07	18.39	1680.22	−20% 54.63	392.4	13.65	28.75
	Standard 1680.22	68.29	392.40	17.07	22.99	1680.22	Standard 68.29	392.4	17.07	22.99
	+20% 2016.26	​	470.88	17.07	27.59	1680.22	+20% 81.95	392.4	20.49	19.15
Finerenone	−20% 1030.26	​	​	​	​	1287.82	−20% 40.98	​	​	​
	Standard 1287.82	51.22	​	​	​	1287.82	Standard 51.22	​	​	​
	+20% 1545.38	​	​	​	​	1287.82	+20% 61.46	​	​	​
Combination	−20% 1344.18	​	506.16	30.44	16.63	1680.22	−20% 55.66	632.7	24.36	25.97
	Standard 1680.22	69.57	632.70	30.44	20.79	1680.22	Standard 69.57	632.7	30.44	20.79
	+20% 2016.26	​	759.24	30.44	24.94	1680.22	+20% 83.48	632.7	36.52	17.32
Dapagliflozin	−20% 838.02	​	​	​	​	1047.52	−20% 31.30	​	​	​
	Standard 1047.52	39.13	​	​	​	1047.52	Standard 39.13	​	​	​
	+20% 1257.02	​	​	​	​	1047.52	+20% 46.96	​	​	​

C, cost; E, effect; ΔC, incremental cost; ΔE, incremental effect; ICER, incremental cost-effectiveness ratio.

Probabilistic sensitivity analysis showed that the median ICER from 1,000 Monte Carlo simulations was 23.26 RMB/% (95% uncertainty interval: −32.05–186.07) for combination vs. finerenone, and 20.82 RMB/% (95% uncertainty interval: −3.71 to 74.53) for combination vs. dapagliflozin, both consistent with the base-case analysis. The probability that combination therapy was superior in terms of remission rate was 94.80% versus finerenone and 99.80% versus dapagliflozin. In 79.70% and 94.60% of simulations, respectively, combination therapy was associated with “higher cost, greater effect” (Supplementary Table S9; Supplementary Figure S1).

### 3.5 Efficacy and safety at different time points

The primary longitudinal analysis was restricted to the 3- to 6-month window using GEE in the full unmatched cohort. For clinical remission, the time effect was significant (Wald χ^2^ = 8.597, *P* = 0.003), while the group effect (*P* = 0.358) and group × time interaction (*P* = 0.594) were not significant. For 24-h urinary protein, both the time effect (Wald χ^2^ = 17.101, *P* = 0.001) and group effect (Wald χ^2^ = 10.175, *P* = 0.006) were significant, while the group × time interaction was not significant (*P* = 0.096). For the rate of reduction in 24-h urinary protein, the time effect was significant (Wald χ^2^ = 18.683, *P* = 0.001), the group effect was marginally significant (Wald χ^2^ = 4.631, *P* = 0.099), and the interaction effect was not significant (*P* = 0.271). For eGFR, the time effect (*P* = 0.928), group effect (*P* = 0.399), and group × time interaction (*P* = 0.306) were all non-significant. To verify the robustness of these findings, a sensitivity GEE analysis including all follow-up time points (3–24 months) in the full unmatched cohort was performed. The group × time interactions for clinical remission, 24-h urinary protein, the rate of reduction in 24-h urinary protein and eGFR were all non-significant (*P* > 0.05), consistent with the primary analysis (Supplementary Tables S10–S17).

In descriptive analyses, from 3 to 24 months, 24-h urinary protein and UACR levels showed sustained downward trends, and the rate of reduction in 24-h urinary protein showed an upward trend in all three groups; eGFR showed an upward trend in the combination group and fluctuating changes in the monotherapy groups. These observations should be considered exploratory and hypothesis-generating, and may be influenced by survivor bias and insufficient statistical power; no conclusions regarding long-term renal stability can be drawn (Supplementary Tables S18, S19; Figure 2).

**FIGURE 2:**
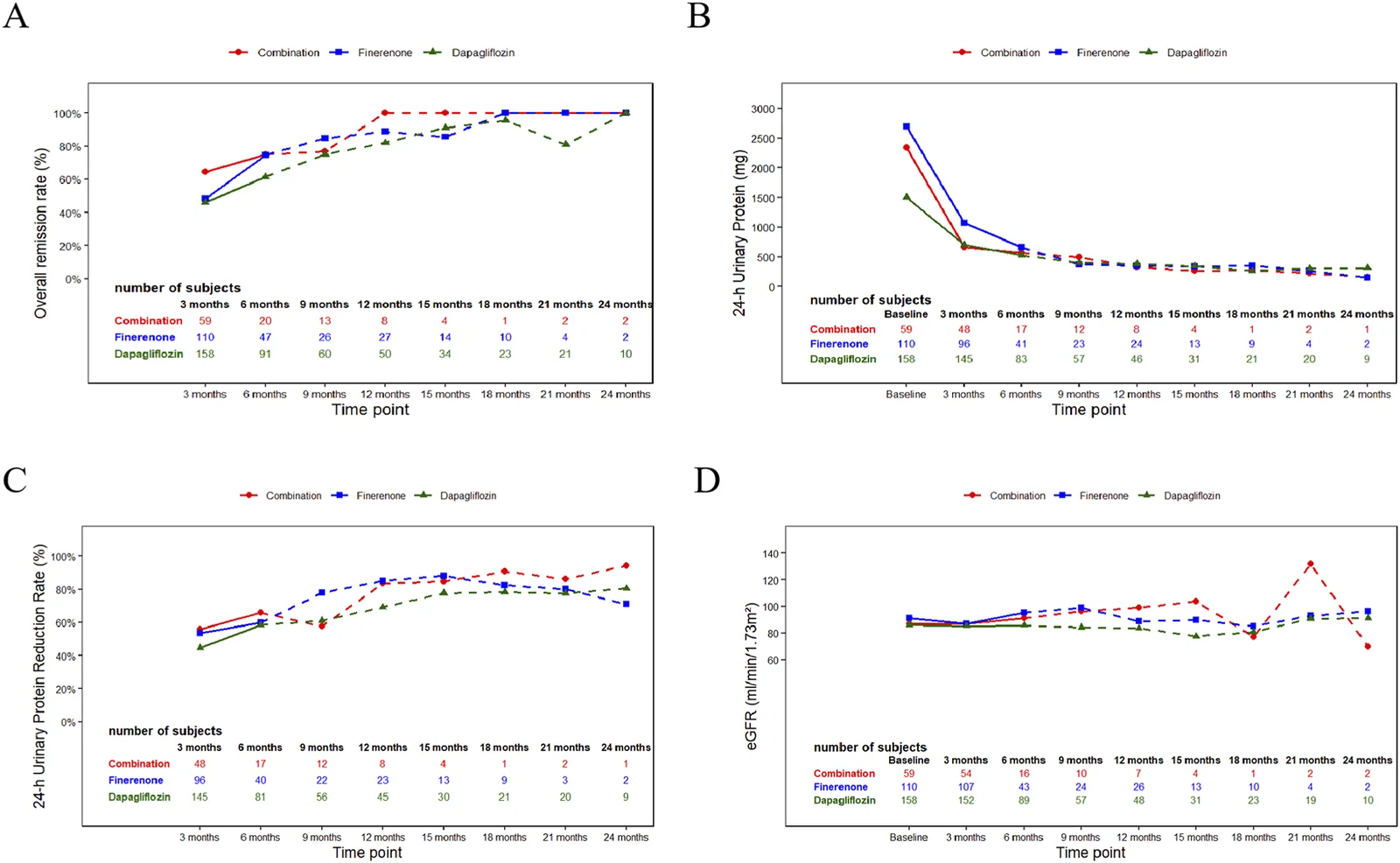
Exploratory descriptive trends in efficacy indicators over time by treatment group (combination therapy, finerenone, and dapagliflozin). **(A)** Overall remission rate, **(B)** 24-h Urinary Protein, **(C)** 24-h Urinary Protein Reduction Rate, **(D)** eGFR.

Regarding safety, adverse event rates were low at all time points, with no obvious increasing trend; however, the long-term safety analysis was underpowered (Supplementary Table S20).

### 3.6 Analysis of influencing factors

Univariate analysis identified treatment regimen, histological type, UACR, 24-h urinary protein, total cholesterol, LDL-C, serum albumin, uric acid, and serum chloride as potential factors associated with treatment outcomes (*P* < 0.1), and these were entered into the multivariate logistic regression model (Table 6). Collinearity diagnostics revealed strong correlations between UACR and 24-h urinary protein (Pearson r = 0.858, *P* < 0.001), and between total cholesterol and LDL-C (Pearson r = 0.933, *P* < 0.001). During variable selection, 24-h urinary protein and UACR both reflect the severity of proteinuria and are highly correlated, as reported in the literature (Visram et al., 2020). Meanwhile, UACR is recommended by KDIGO guidelines as the preferred indicator for proteinuria assessment (KDIGO, 2024), and LDL-C is the core therapeutic target for lipid management (Zhu et al., 2016); thus, UACR and LDL-C were retained. The final model included treatment regimen, histological type, UACR, serum albumin, LDL-C, uric acid, and serum chloride.

**TABLE 6:** Univariate analysis of treatment failure in the full unmatched cohort (n = 327).

Variable	Total (n = 327)	Treatment success (n = 164)	Treatment failure (n = 163)	t/Z/X^2^	*P*
Age, years	48.00 (36.00, 60.00)	47.00 (36.00, 59.00)	48.00 (36.00, 60.00)	−0.799	0.424
Sex, male, n (%)	181 (55.35)	89 (54.27)	92 (56.44)	0.156	0.693
Weight, kg	66.00 (59.80, 75.00)	65.10 (58.08, 75.63)	66.00 (60.00, 75.00)	−0.517	0.605
BMI, kg/m^2^	24.32 (22.68, 26.81)	24.39 (22.76, 26.96)	24.32 (22.64, 26.68)	−0.742	0.458
SBP, mmHg	129.00 (120.00, 139.00)	128.00 (119.25, 138.00)	130.00 (120.00, 140.00)	−1.129	0.259
DBP, mmHg	82.00 (75.00, 89.00)	82.00 (75.00, 88.75)	82.00 (77.00, 90.00)	−1.215	0.224
Treatment regimen	​	​	​	5.952	0.051
Finerenone	110 (33.64)	53 (32.32)	57 (34.97)	​	​
Dapagliflozin	158 (48.32)	73 (44.51)	85 (52.15)	​	​
Combination	59 (18.04)	38 (23.17)	21 (12.88)	​	​
Histological type	​	​	​	8.145	0.043
Membranous nephropathy, n (%)	156 (47.71)	69 (42.07)	87 (53.37)	​	​
IgA nephropathy, n (%)	123 (37.61)	65 (39.63)	58 (35.58)	​	​
Primary glomerular disease, n (%)	15 (4.59)	12 (7.32)	3 (1.84)	​	​
Secondary/Other nephropathy, n (%)	33 (10.09)	18 (10.98)	15 (9.20)	​	​
Hypertension, n (%)	182 (55.66)	92 (56.10)	90 (55.21)	0.026	0.872
Hyperlipidemia, n (%)	143 (43.73)	74 (45.12)	69 (42.33)	0.259	0.611
24-h urine volume, ml	2000.00 (1600.00, 2500.00)	2000.00 (1600.00, 2550.00)	2000.00 (1500.00, 2500.00)	−1.163	0.245
UACR, mg/g	932.30 (396.10, 2256.80)	774.30 (387.83, 1888.98)	1210.60 (435.70, 2845.70)	−2.253	0.024
24-h urinary protein, mg	1764.00 (925.00, 3786.00)	1622.50 (835.00, 3484.75)	2080.00 (954.00, 4468.00)	−1.806	0.071
eGFR, mL·min^−1^·1.73 m^−2^	87.85 ± 31.56	88.31 ± 32.74	87.39 ± 30.41	−0.263	0.793
Scr, µmol/L	79.00 (64.00, 104.00)	79.00 (50.50, 105.50)	79.00 (55.40, 103.00)	−0.196	0.845
CRP, mg/L	3.80 (2.60, 5.90)	3.80 (2.53, 6.43)	3.80 (2.60, 5.80)	−0.343	0.731
Triglycerides, mmol/L	1.67 (1.12, 2.46)	1.66 (1.10, 2.23)	1.68 (1.19, 2.55)	−1.336	0.182
Total cholesterol, mmol/L	5.26 (4.46, 6.14)	5.14 (4.32, 5.95)	5.38 (4.63, 6.49)	−2.464	0.014
HDL-C, mmol/L	1.33 (1.11, 1.58)	1.32 (1.08, 1.53)	1.33 (1.13, 1.63)	−0.898	0.369
LDL-C, mmol/L	2.97 (2.49, 3.59)	2.94 (2.38, 3.48)	3.01 (2.61, 3.81)	−1.793	0.073
Platelet count, ×10^9^/L	227.00 (198.00, 251.00)	227.00 (203.00, 261.75)	227.00 (196.00, 248.00)	−0.317	0.751
Hb, g/L	135.00 (124.00, 148.00)	135.00 (123.00, 158.50)	135.00 (128.00, 164.50)	−0.836	0.403
Alkaline phosphatase, U/L	66.70 (57.60, 79.60)	66.30 (57.90, 80.28)	66.70 (57.10, 79.50)	−0.102	0.918
Serum albumin, g/L	38.70 (32.90, 42.40)	39.50 (34.63, 43.05)	37.10 (30.70, 41.90)	−2.583	0.010
Uric acid, µmol/L	342.00 (289.00, 405.00)	337.00 (280.25, 384.75)	357.00 (301.00, 427.00)	−2.146	0.032
Serum phosphorus, mmol/L	1.20 (1.08, 1.32)	1.20 (1.09, 1.29)	1.20 (1.08, 1.34)	−0.652	0.515
Serum potassium, mmol/L	4.03 (3.79, 4.24)	4.05 (3.61, 4.31)	4.03 (3.76, 4.23)	−1.139	0.255
Serum sodium, mmol/L	139.50 (138.10, 141.10)	139.35 (136.70, 140.90)	139.70 (138.20, 141.30)	−1.291	0.197
Serum chloride, mmol/L	105.70 (103.80, 107.40)	105.90 (101.95, 107.75)	105.90 (104.00, 107.80)	−1.801	0.072
Serum bicarbonate, mmol/L	25.84 ± 2.74	25.91 ± 2.62	25.77 ± 2.85	−0.482	0.630

Abbreviations as in Table 1. Additional abbreviations: Scr, serum creatinine; CRP, C-reactive protein; HDL-C, high-density lipoprotein cholesterol; LDL-C, low-density lipoprotein cholesterol; Hb, hemoglobin.

As shown in Table 7, dapagliflozin monotherapy was an independent risk factor for treatment failure. Compared with combination therapy, dapagliflozin monotherapy was associated with an approximately 2.16-fold increased risk of treatment failure (OR = 2.163, 95% CI: 1.073–4.362, *P* = 0.031). Finerenone monotherapy did not reach statistical significance compared with combination therapy (OR = 1.647, 95% CI: 0.814–3.334, *P* = 0.165). Regarding nephropathy type, with MN as the reference, primary glomerular disease was significantly associated with a lower risk of treatment failure (OR = 0.209, 95% CI: 0.052–0.844, *P* = 0.028), suggesting that patients with primary glomerular disease responded better to the therapeutic regimens than those with MN. No significant differences were observed for IgA nephropathy or secondary/other nephropathy compared with MN (*P* > 0.05). Additionally, elevated baseline uric acid level emerged as a marginal risk factor for treatment failure (OR = 1.002, 95% CI: 1.000–1.005, *P* = 0.060). Other variables, including UACR, serum albumin, LDL-C, and serum chloride, were not significantly associated with treatment failure (*P* > 0.05).

**TABLE 7:** Multivariate logistic regression analysis of treatment failure in the full unmatched cohort (n = 327).

Variable	β	S.E.	Wald	OR	95% CI	*P*
Treatment regimen (ref: Combination)						
Finerenone vs. combination	0.499	0.360	1.925	1.647	0.814–3.334	0.165
Dapagliflozin vs. combination	0.771	0.358	4.646	2.163	1.073–4.362	0.031
Nephropathy type (ref: MN)						
IgA nephropathy vs. MN	−0.101	0.304	0.111	0.904	0.499–1.639	0.739
Primary glomerular disease vs. MN	−1.563	0.711	4.834	0.209	0.052–0.844	0.028
Secondary/Other nephropathy vs. MN	−0.239	0.415	0.332	0.787	0.349–1.775	0.564
UACR, mg/g	0.000	0.000	1.860	1.000	1.000–1.000	0.173
Serum albumin, g/L	−0.022	0.026	0.704	0.979	0.930–1.029	0.402
LDL-C, mmol/L	0.000	0.124	0.000	1.000	0.784–1.274	0.997
Uric acid, µmol/L	0.002	0.001	3.533	1.002	1.000–1.005	0.060
Serum chloride, mmol/L	0.009	0.025	0.128	1.009	0.961–1.060	0.721

Dependent variable: treatment failure (failure = 1, success = 0). Combination therapy and MN, were set as the reference groups. Abbreviations as in Table 1. Additional abbreviations: LDL-C, low-density lipoprotein cholesterol; OR, odds ratio; CI, confidence interval.

This multivariate logistic regression was performed in the pre-PSM full cohort (n = 327) for exploratory risk-factor identification; these results should not be interpreted as a validated clinical prediction model.

### 3.7 Subgroup analysis

Given that the risk factor analysis suggested a relatively higher risk of treatment failure in patients with MN, we further performed exploratory subgroup analyses in patients with MN and hypertension identified from the post-PSM cohort.

MN subgroup: Baseline characteristics among the three groups after PSM are shown in Supplementary Table S21. The overall remission rates in the combination group (n = 21) and finerenone group (n = 21) were 71.43% and 42.86%, respectively (OR = 3.33, 95% CI: 0.93–12.05; *P* = 0.061), without statistically significant difference. The overall remission rates in the combination group (n = 30) and dapagliflozin group (n = 20) were 63.33% and 30.00%, respectively (OR = 4.03, 95% CI: 1.20–13.51; *P* = 0.021), with a statistically significant difference (Table 8). For 24-h urinary protein, the combination group had significantly lower levels than both the finerenone group (*P* = 0.006) and the dapagliflozin group (*P* = 0.045). No statistically significant differences were observed in other primary efficacy outcomes, secondary efficacy outcomes or safety outcomes among groups (*P* > 0.05) (Table 9; Supplementary Tables S22, S23).

**TABLE 8:** Membranous nephropathy subgroups: comparison of clinical efficacy at 3 months among the three groups after PSM.

Response criteria	Combination (n = 21)	Finerenone (n = 21)	OR (95% CI)	*P*	Combination (n = 30)	Dapagliflozin (n = 20)	OR (95% CI)	*P*
Overall response rate (%)	15 (71.43)	9 (42.86)	3.33 (0.93–12.05)	0.061	19 (63.33)	6 (30.00)	4.03 (1.20–13.51)	0.021
Complete remission (%)	4 (19.05)	1 (4.76)	​	​	6 (20.00)	1 (5.00)	​	​
Partial remission (%)	11 (52.38)	8 (38.10)	​	​	13 (43.33)	5 (25.00)	​	​
No response (%)	6 (28.57)	12 (57.14)	​	​	11 (36.67)	14 (70.00)	​	​

OR, odds ratio; CI, confidence interval.

**TABLE 9:** Membranous nephropathy subgroups: comparison of primary efficacy outcomes at 3 months among the three groups after PSM.

Variable	Combination (n = 21)	Finerenone (n = 21)	t/Z/X^2^	*P*	Combination (n = 30)	Dapagliflozin (n = 20)	t/Z/X^2^	*P*
24-h urine volume, ml	1975.00 (1525.00, 2400.00)	1500.00 (1000.00, 2500.00)	−1.168	0.243	2000.00 (1550.00, 2500.00)	2115.00 (1900.00, 2740.00)	−0.778	0.437
UACR, mg/g	359.40 (123.75, 1005.10)	1146.35 (410.48, 1766.30)	−1.930	0.054	409.00 (109.15, 2226.38)	690.00 (218.50, 4075.40)	−1.171	0.242
24-h urinary protein, mg	799.00 (554.00, 1155.00)	2501.00 (1019.75, 5203.25)	−2.735	0.006	856.50 (471.25, 1985.75)	2198.50 (787.00, 4997.75)	−2.003	0.045
eGFR, mL·min^−1^·1.73 m^−2^	81.30 ± 29.19	86.94 ± 29.95	0.603	0.550	95.10 (64.13, 103.95)	95.05 (79.53, 111.25)	−0.795	0.427
Scr, µmol/L	82.00 (72.00, 108.00)	86.00 (65.50, 100.50)	−0.257	0.797	80.50 (68.25, 100.25)	71.50 (60.25, 78.75)	−1.821	0.069
Δ SBP (%)	−6.37 ± 11.70	−2.75 ± 13.14	0.618	0.545	−3.01 ± 12.83	−10.74 ± 14.69	−1.565	0.128
Δ DBP (%)	−5.85 ± 17.84	−8.95 ± 12.48	−0.415	0.684	−0.40 ± 19.52	−11.43 ± 23.46	−1.430	0.164
Δ UACR (%)	65.76 (41.79, 81.07)	66.80 (40.54, 80.35)	−0.209	0.835	61.26 (37.64, 78.45)	51.54 (27.48, 66.05)	−1.058	0.290
Δ 24-h urinary protein (%)	58.56 (51.38, 74.51)	54.15 (36.33, 65.42)	−1.307	0.191	54.41 ± 26.72	46.71 ± 20.82	−1.035	0.306
Δ eGFR (%)	−2.29 (−7.92, 4.22)	−3.35 (−10.04, 9.39)	−0.122	0.903	−0.08 ± 9.73	2.59 ± 18.77	0.584	0.564
Δ Scr (%)	1.25 ± 10.18	3.03 ± 14.39	0.447	0.657	−0.63 (−6.82, 5.75)	−2.53 (−12.74, 11.57)	−0.366	0.714

Δ, percent change from baseline; abbreviations as in Table 1. Additional abbreviations: Scr, serum creatinine.

Hypertension subgroup: Baseline characteristics among the three groups after PSM are shown in Supplementary Table S24. The overall remission rates in the combination group (n = 27) and finerenone group (n = 24) were 62.96% and 54.17%, respectively (OR = 1.44, 95% CI: 0.47–4.41; *P* = 0.524), without statistical significance. The overall remission rates in the combination group (n = 31) and dapagliflozin group (n = 24) were 70.97% and 33.33%, respectively (OR = 4.88, 95% CI: 1.55–15.38; *P* = 0.005), with a statistically significant difference (Table 10). No statistically significant differences were observed in primary efficacy outcomes, secondary efficacy outcomes or safety outcomes among groups (*P* > 0.05) (Table 11; Supplementary Tables S25, S26).

**TABLE 10:** Hypertension subgroups: comparison of clinical efficacy at 3 months among the three groups after PSM.

Response criteria	Combination (n = 27)	Finerenone (n = 24)	OR (95% CI)	*P*	Combination (n = 31)	Dapagliflozin (n = 24)	OR (95% CI)	*P*
Overall response rate (%)	17 (62.96)	13 (54.17)	1.44 (0.47–4.41)	0.524	22 (70.97)	8 (33.33)	4.88 (1.55–15.38)	0.005
Complete remission (%)	6 (22.22)	7 (29.17)	​	​	7 (22.58)	2 (8.33)	​	​
Partial remission (%)	11 (40.74)	6 (25.00)	​	​	15 (48.39)	6 (25.00)	​	​
No response (%)	10 (37.04)	11 (45.83)	​	​	9 (29.03)	16 (66.67)	​	​

OR, odds ratio; CI, confidence interval.

**TABLE 11:** Hypertension subgroups: comparison of primary efficacy outcomes at 3 months among the three groups after PSM.

Variable	Combination (n = 27)	Finerenone (n = 24)	t/Z/X^2^	*P*	Combination (n = 31)	Dapagliflozin (n = 24)	t/Z/X^2^	*P*
24-h urine volume, ml	1800.00 (1425.00, 2400.00)	1850.00 (1175.00, 2725.00)	−0.076	0.939	1925.00 (1425.00, 2475.00)	1930.00 (1500.00, 2175.00)	−0.167	0.868
UACR, mg/g	293.00 (90.50, 539.50)	358.50 (115.00, 783.50)	−0.594	0.553	283.00 (87.50, 1017.85)	646.70 (157.90, 1728.80)	−1.338	0.181
24-h urinary protein, mg	655.00 (433.00, 954.00)	678.00 (286.00, 1175.00)	−0.265	0.791	619.00 (343.00, 1155.00)	783.50 (466.50, 3158.25)	−1.256	0.209
eGFR, mL·min^−1^·1.73 m^−2^	76.53 ± 32.45	70.67 ± 36.98	−0.577	0.567	81.59 ± 31.94	80.34 ± 32.18	−0.139	0.890
Scr, µmol/L	93.00 (70.00, 121.50)	98.50 (77.00, 139.25)	−0.770	0.441	88.50 (68.00, 117.00)	81.00 (64.25, 105.00)	−0.606	0.545
Δ SBP (%)	−3.56 ± 12.81	−3.54 ± 11.78	0.004	0.997	−2.28 ± 11.35	−6.52 ± 19.25	−0.763	0.452
Δ DBP (%)	−5.51 ± 16.36	−4.55 ± 11.44	0.151	0.881	−1.67 (−15.27, 3.33)	−8.49 (−23.97, 0.00)	−1.036	0.300
Δ UACR (%)	55.97 ± 24.69	55.25 ± 29.96	−0.094	0.926	57.75 ± 24.50	47.09 ± 34.30	−1.244	0.220
Δ 24-h urinary protein (%)	55.22 ± 23.27	45.40 ± 30.67	−1.179	0.245	54.93 ± 22.63	49.87 ± 21.07	−0.804	0.425
Δ eGFR (%)	−1.41 ± 10.39	3.41 ± 19.20	1.065	0.295	−1.10 ± 11.40	−2.12 ± 18.88	−0.236	0.815
Δ Scr (%)	1.09 (−7.09, 8.15)	−6.47 (−13.29, 6.44)	−1.210	0.226	1.14 (−8.76, 9.41)	1.98 (−8.34, 12.28)	−0.477	0.633

Δ, percent change from baseline; abbreviations as in Table 1. Additional abbreviations: Scr, serum creatinine.

## 4 Discussion

Finerenone, a third-generation nonsteroidal mineralocorticoid receptor antagonist (nsMRA), primarily alleviates glomerulosclerosis and tubulointerstitial damage by blocking the inflammatory and fibrotic pathways mediated by excessive MR activation. In contrast, dapagliflozin as SGLT2i, exerts its renoprotective effects through multiple mechanisms, including restoration of the tubuloglomerular feedback loop, reduction of glomerular pressure, and improvement of renal oxygenation and metabolic homeostasis (Hu Y. et al., 2025). Theoretically, the combination of these two agents may produce a synergistic effect in reducing proteinuria and slowing the decline in renal function. A review by Asirvatham et al. noted that the combination of SGLT2i and finerenone, through a dual-pathway intervention involving “hemodynamic effects plus anti-inflammatory and antifibrotic effects,” provides a theoretical basis for the precision treatment of DKD and NDKD (Asirvatham et al., 2024). Based on real-world data, this study employed PSM to systematically evaluate the clinical value of finerenone combined with dapagliflozin in the treatment of NDKD.

The results showed that after PSM, at 3 months of treatment, the overall remission rates in the combination and finerenone groups were 68.29% and 51.22%, respectively (OR = 2.05, 95% CI: 0.84–5.05, *P* = 0.115). The overall remission rates in the combination and dapagliflozin groups were 69.57% and 39.13%, respectively (OR = 3.56, 95% CI: 1.50–8.40, *P* = 0.003). In the sensitivity analysis, the direction of the treatment effect was consistent with the primary analysis. Combination therapy remained superior to dapagliflozin monotherapy in terms of overall remission rate, whereas the comparison with finerenone monotherapy did not reach statistical significance. Moreover, the reductions in 24-h urinary protein and the elevation in serum albumin were higher in the combination group. This short-term therapeutic advantage can be explained by the complementary mechanisms of action of the two drugs. These findings are consistent with those of the first randomized controlled trial by Mårup et al. (2024), which demonstrated that 8 weeks of combination therapy with finerenone and dapagliflozin significantly reduced urinary albumin levels in patients with NDKD, providing significant early benefits and showing a synergistic effect superior to the sum of the individual drug effects. In the primary 3- to 6-month GEE analysis, time effects were significant for remission and proteinuria. For 24-h urinary protein, the group effect was also significant (*P* = 0.006), indicating significant differences among the three groups in proteinuria levels during this early window; however, the group × time interaction was not significant, suggesting that the rate of improvement did not differ significantly between groups. For the rate of reduction in 24-h urinary protein, the group effect was marginally significant (*P* = 0.099). These findings are directionally consistent with a study by Ma et al. (2026), which reported that in patients with NDKD, a 6-month regimen of finerenone combined with dapagliflozin led to a significant reduction in albuminuria after 3–6 months of standardized intervention. A sensitivity GEE analysis including all follow-up time points (3–24 months) yielded consistent results, with non-significant group × time interactions for clinical remission, 24-h urinary protein, and its rate of reduction. In exploratory descriptive analyses, from 3 to 24 months, clinical remission rates, 24-h urinary protein levels, and the rate of reduction in 24-h urinary protein all showed numerical improvement trends across the three groups: 24-h urinary protein levels continued to decline, its rate of reduction gradually increased, and clinical remission rates numerically increased over time. Beyond 6 months, sample sizes declined substantially, and data from 9 to 24 months are descriptive only, without formal statistical comparisons. These observations should be considered exploratory and hypothesis-generating, and the apparent convergence may reflect survivor bias and insufficient statistical power rather than true equivalence.

For eGFR, time, group, and interaction effects were all non-significant. No significant decline in eGFR was observed through 12 months in the available cohort; data beyond 12 months were too sparse for reliable inference. eGFR in the combination group showed a numerical upward trend, which may be related to resolution of glomerular hyperfiltration following albuminuria reduction, or may be influenced by survivor bias during follow-up. These observations are exploratory and hypothesis-generating, and no conclusions regarding long-term renal stability can be drawn. The short-term (3–6 months) findings of this study are directionally consistent with the eGFR-slowing effect of dapagliflozin in the DAPA-CKD trial and the renal protective effect of finerenone in the FIDELIO-DKD trial (Bakris et al., 2020; Heerspink et al., 2020), but long-term renal outcomes require validation in larger prospective studies.

Another key finding of this study relates to safety. In the available follow-up data, no statistically significant between-group differences in overall adverse event rates were detected. Long-term safety is a core consideration in the maintenance treatment of NDKD. Dapagliflozin as SGLT2i, induces osmotic diuresis and glycosuria; its most common adverse reactions include genitourinary fungal infections. Finerenone, as a nonsteroidal mineralocorticoid receptor antagonist, reduces renal potassium excretion by blocking the MR; its most common adverse reaction is hyperkalemia (Heerspink et al., 2016; Agarwal et al., 2022). A meta-analysis by Yuan et al. (2024) found that SGLT2i, by inducing osmotic diuresis, increasing distal sodium delivery, and promoting potassium excretion, can significantly reduce the risk of hyperkalemia caused by MRAs, and that the combination of the two drugs exhibits a synergistic effect with a favorable safety profile. In this study, no statistically significant differences in the incidence of adverse reactions were observed among the three groups before or after PSM, whether at the short-term (3 months) follow-up or at the medium- to long-term follow-ups (6–24 months). Adverse events were primarily mild hyperkalemia and genitourinary infections. However, because the number of events was very small and the analysis was underpowered, these findings cannot exclude an increased risk of serious adverse events or other safety concerns associated with combination therapy. A retrospective real-world study by Albakr et al. (2025) on finerenone in patients with NDKD also confirmed that finerenone monotherapy significantly reduces albuminuria, maintains stable serum potassium levels throughout the treatment course, and is associated with no cases of severe hyperkalemia or adverse cardiac or renal events, these findings are consistent with the safety results of the present study. The safety analysis of the CONFIDENCE trial provides important external validation for this finding. The incidence of hyperkalemia was 9.3% in the combination therapy group, compared with 11.4% in the finerenone monotherapy group, suggesting that the addition of an SGLT2i may mitigate the risk of finerenone-induced hyperkalemia to some extent (Agarwal et al., 2025). A real-world study by Hanouneh et al. (2024) similarly reported that combination therapy did not increase the risk of hyperkalemia. Taken together, the available evidence suggests that combination therapy was associated with low observed adverse event rates during the available follow-up. However, because the number of events was small and follow-up beyond 6 months was limited, these findings cannot establish that long-term combination therapy is well tolerated or that its adverse event risk is equivalent to that of monotherapy. Long-term safety remains uncertain and requires confirmation in larger prospective studies with adequate statistical power.

Univariate and multivariate logistic regression analyses (based on the pre-PSM full cohort, n = 327) showed that, dapagliflozin monotherapy was independently associated with an increased risk of treatment failure (OR = 2.163, 95% CI: 1.073–4.362, *P* = 0.031), corresponding to an approximately 2.16-fold higher risk compared with combination therapy. Hanouneh et al. (2024) reported that, among the 24 patients receiving combination therapy, 96% achieved a >50% reduction in proteinuria within 8 months, compared with 50% of those receiving dapagliflozin monotherapy and 59% of those receiving finerenone monotherapy. In patients with NDKD who require substantial proteinuria reduction, dapagliflozin monotherapy may not suffice to achieve optimal therapeutic control, whereas combination therapy with finerenone may reduce the risk of treatment failure.

Regarding nephropathy type, with membranous nephropathy (MN) as the reference, primary glomerular disease was associated with a lower risk of treatment failure (OR = 0.209, 95% CI: 0.052–0.844, *P* = 0.028), suggesting that patients with primary glomerular disease may respond better to the therapeutic regimens in this study. These findings suggest that different pathological types of NDKD may exhibit heterogeneity in response to the same treatment regimen, with MN patients showing a relatively higher risk of treatment failure, warranting further exploration of whether more aggressive combination therapy is needed for this subgroup.

Of note, this multivariate logistic regression was performed in the pre-PSM full cohort (n = 327) for exploratory risk-factor identification; the results reflect associations rather than causal relationships and have not been externally validated. These findings, including the subgroup differences across nephropathy types, should be considered exploratory and warrant further validation in larger prospective studies. To preliminarily explore the above questions, we further evaluated the efficacy of different treatment regimens in the membranous nephropathy subgroup.

Membranous nephropathy is one of the most common pathological types of NDKD and also one of the most common causes of primary nephrotic syndrome in adults, it is caused by the deposition of antigen-antibody complexes between the glomerular basement membrane (GBM) and podocytes and is typically characterized by massive proteinuria and hypoproteinemia (Alok and Yadav, 2023). Finerenone reduces proteinuria in MN by mitigating podocyte damage through its anti-inflammatory, antifibrotic, and antioxidant effects (Cong et al., 2025). On the other hand, dapagliflozin further reduces proteinuria by lowering intraglomerular pressure (Dekkers et al., 2018). Therefore, combination therapy with finerenone and dapagliflozin may be more suitable for this patient population. Meanwhile, in the risk factor analysis, using MN as the reference, primary glomerular disease was associated with a lower risk of treatment failure, suggesting that patients with MN may have a relatively higher risk of treatment failure. Therefore, we further evaluated the efficacy of different regimens in the MN subgroup. The results showed that in the MN subgroup, combination therapy significantly improved the short-term overall remission rate compared with dapagliflozin monotherapy (OR = 4.03, *P* = 0.021) and significantly reduced 24-h urinary protein levels (*P* = 0.045). Compared with finerenone monotherapy, the combination group had a numerically higher remission rate (71.43% vs. 42.86%), but the difference did not reach statistical significance (*P* = 0.061), possibly due to limited sample size and statistical power. These findings further support the observations from the risk factor analysis, suggesting that MN patients may derive greater short-term efficacy benefits from combination therapy, but should be interpreted with caution.

Finerenone blocks MR in the kidneys, directly inhibiting aldosterone-induced sodium reabsorption, thereby promoting water and sodium excretion and exerting a blood pressure-lowering effect (Kolkhof et al., 2022). SGLT2i reduce plasma volume through natriuretic and diuretic effects and exert their blood pressure-lowering effects by improving vascular endothelial function (Wilcox, 2020). A meta-analysis by Ferreira et al. further confirmed that, compared with SGLT2i monotherapy, the combination of a MRA and an SGLT2i resulted in an additional reduction in systolic blood pressure of 6.1 mmHg (95% CI: −8.9 to −3.3 mmHg, *P* < 0.001), providing evidence-based support for the use of this combination therapy in patients with hypertension and CKD (Ferreira et al., 2025). In the hypertension subgroup, combination therapy also showed a significantly higher overall remission rate compared with dapagliflozin monotherapy (OR = 4.88, *P* = 0.005), whereas the difference compared with finerenone monotherapy was not statistically significant (*P* = 0.524). No significant differences in blood pressure changes were observed among groups, suggesting that the potential advantage of combination therapy in the hypertension subgroup may not depend primarily on further blood pressure reduction. These subgroup analyses are exploratory and limited by small sample sizes; the results require validation in larger prospective studies.

The cost-effectiveness analysis showed that, although the combination therapy regimen incurred higher treatment costs, it demonstrated a reasonable ICER. Compared with finerenone monotherapy, each 1% increase in the clinical remission rate was associated with an additional cost of approximately 22.99 RMB. Compared with dapagliflozin monotherapy, each 1% increase in remission rate was associated with an additional cost of approximately 20.79 RMB. Both one-way sensitivity analysis (±20%) and probabilistic sensitivity analysis (1,000 Monte Carlo simulations) showed stable ICER ranges consistent with the base-case analysis, indicating robust model findings. A study found that triple therapy with finerenone, an SGLT2i, and standard of care (SoC)—i.e., finerenone + SGLT2i + SoC—offered superior cost-effectiveness in patients with type 2 diabetes and chronic kidney disease compared with adding either finerenone or an SGLT2i as an adjunct to SoC (Hu Z. et al., 2025). Although combination therapy incurs higher short-term medication costs, the incremental short-term expenditure can be regarded as the “time value” of achieving rapid control of proteinuria and improvement of clinical symptoms. Furthermore, this exploratory 3-month analysis cannot determine long-term cost-effectiveness; future long-term economic evaluations are needed.

Of note, this cost analysis is based on 3-month short-term data and includes only direct medical costs, without accounting for indirect costs or health benefits from long-term prognosis improvement. Therefore, this analysis is exploratory and descriptive, and no judgment of economic superiority or inferiority is made.

This study has the following limitations. First, the follow-up duration was confounded with calendar time of enrollment, because patients enrolled before June 2022 all received dapagliflozin monotherapy and finerenone-containing regimens became available only after June 2022. As a result, long-term follow-up data were unbalanced across groups. Therefore, data beyond 6 months are presented descriptively only, without between-group inferential comparisons, and should not be interpreted as evidence of long-term efficacy comparison or therapeutic equivalence; Second, as a retrospective cohort study, although PSM was used to balance baseline confounding factors, the influence of unmeasured confounders cannot be completely ruled out. Clinicians tended to select combination therapy for patients with higher baseline proteinuria or higher renal risk; Additionally, clinical practice varied, and not all patients achieved maximally tolerated RAAS inhibitor doses before initiating finerenone or dapagliflozin, which may have influenced treatment outcomes and represents an important confounding factor in real-world retrospective research; Third, the economic analysis employed a simplified model that included only direct medical costs and did not account for indirect costs or the health benefits derived from long-term improvements in prognosis, so this analysis is exploratory and descriptive, and no judgment of economic superiority or inferiority is made. Fourth, the multivariate regression analysis was based on the full unmatched cohort and aimed at exploratory risk-factor identification; it was not designed or validated as a predictive model for individualized clinical prediction. Future studies could include multicenter, prospective controlled trials with follow-up periods extending beyond 3 years, following the design of the FIND-CKD trial, to further validate the long-term renal and cardiovascular benefits of combination therapy. In parallel, subgroup analyses could be performed according to different pathological types and eGFR stages to optimize individualized dosing and treatment duration, thereby providing higher-level evidence to support the development of a standardized diagnostic and treatment system for NDKD.

## 5 Conclusion

Finerenone combined with dapagliflozin provides higher observed short-term (3-month) efficacy in NDKD, with low observed adverse event rates. Exploratory analyses support prioritizing dual therapy for rapid disease control in high-risk phenotypes, while monotherapy with structured monitoring remains an acceptable alternative for stable patients. Long-term outcomes require validation in larger prospective studies.

## Data Availability

The raw data supporting the conclusions of this article will be made available by the authors, without undue reservation.
