# Supplementary material for: Short-term superiority in proteinuria control with exploratory longitudinal trajectories: finerenone-dapagliflozin combination in non-diabetic kidney disease
Source: Front Pharmacol. 2026 Oct 13;17:1918911. doi: 10.3389/fphar.2026.1918911 (PMC13710229; doi:10.3389/fphar.2026.1918911)
Supplement: Supplementary file 1 [file Supplementaryfile1.docx]

***Supplementary Material***

**Supplementary Table S1.** Comparison of baseline characteristics among the three groups before PSM

| **Variable** | **Combination**  **(n=59)** | **Finerenone**  **(n=110)** | **t/Z/X^2^** | ***P*** | **Dapagliflozin**  **(n=158)** | **t/Z/X^2^** | ***P*** |
| --- | --- | --- | --- | --- | --- | --- | --- |
| **Age, years** | **47.00(36.00,66.00)** | **50.00(38.00,60.25)** | **-0.066** | **0.947** | **47.00(35.75,57.00)** | **-1.031** | **0.303** |
| **Sex, male, n (%)** | **39(66.10)** | **59(53.64)** | **2.449** | **0.118** | **83(52.5)** | **3.214** | **0.073** |
| **Weight, kg** | **70.00(60.50,77.90)** | **65.00(59.68,74)** | **-1.953** | **0.051** | **66.00(57.98,75.85)** | **-1.551** | **0.121** |
| **BMI, kg/m^2^** | **24.39(23.16,27.34)** | **24.28(22.53,26.31)** | **-0.961** | **0.336** | **24.32(22.59,26.77)** | **-0.916** | **0.359** |
| **SBP, mmHg** | **122.00(115.00,134.00)** | **135(127.75,140.00)** | **-4.650** | **<0.001** | **128.00(118.75,139.00)** | **-1.932** | **0.053** |
| **DBP, mmHg** | **75.00(71.00,85.00)** | **84.00(79.00,90.00)** | **-5.025** | **<0.001** | **82.00(75.75,89.25)** | **-3.615** | **<0.001** |
| **Histological type** |  |  | **6.869** | **0.074** |  | **46.770** | **<0.001** |
| **Membranous nephropathy, n (%)** | **31(52.54)** | **65(59.09)** |  |  | **60(38.00)** |  |  |
| **IgA nephropathy, n (%)** | **10(16.95)** | **20(18.18)** |  |  | **93(58.90)** |  |  |
| **Primary glomerular disease, n (%)** | **9(15.25)** | **4(3.64)** |  |  | **2(1.27)** |  |  |
| **Secondary / Other nephropathy, n (%)** | **9(15.25)** | **21(19.09)** |  |  | **3(1.90)** |  |  |
| **Hypertension, n (%)** | **37(62.71)** | **63(57.27)** | **0.470** | **0.493** | **82(51.90)** | **2.028** | **0.154** |
| **Medication** |  |  |  |  |  |  |  |
| **ACEI, n (%)** | **0(0.00)** | **3(2.73)** |  |  | **2(1.27)** |  |  |
| **ARB, n (%)** | **25(42.37)** | **27(24.55)** |  |  | **51(32.28)** |  |  |
| **Calcium channel blocker, n (%)** | **19(32.20)** | **27(24.55)** |  |  | **38(24.05)** |  |  |
| **Diuretic, n (%)** | **4(6.78)** | **4(3.64)** |  |  | **4(2.53)** |  |  |
| **β‑blocker, n (%)** | **6(10.17)** | **11(10.00)** |  |  | **14(8.86)** |  |  |
| **α‑blocker, n (%)** | **0(0.00)** | **2(1.82)** |  |  | **2(1.27)** |  |  |
| **Centrally acting antihypertensive, n (%)** | **0(0.00)** | **2(1.82)** |  |  | **0(0.00)** |  |  |
| **Hyperlipidemia, n (%)** | **18(30.51)** | **54(49.09)** | **5.423** | **0.020** | **71(44.90)** | **3.6978** | **0.055** |
| **24‑hour urine volume, ml** | **2100.00(1800.00,2550.00)** | **1900.00(1292.50,2212.50)** | **-3.363** | **<0.001** | **2140.00(1600.00,2562.50)** | **-0.496** | **0.620** |
| **UACR, mg/g** | **1183.80(390.40,3631.80)** | **1536.80(499.08,3013.43)** | **-0.640** | **0.522** | **774.30(328.70,1346.50)** | **-2.066** | **0.039** |
| **24‑hour urinary protein, mg** | **2339.00(1030.00,4296.00)** | **2696.00(939.00,5038.75)** | **-0.284** | **0.777** | **1504.00(801.75,2725.25)** | **-2.227** | **0.026** |
| **eGFR, mL·min^-1^·1.73 m^-2^** | **87.40(62.10,105.40)** | **94.70(64.70,118.85)** | **-1.017** | **0.309** | **82.70(66.63,101.20)** | **-0.332** | **0.740** |
| **Scr, µmol/L** | **82.00(69.00,104.00)** | **73.00(59.50,109.25)** | **-1.481** | **0.139** | **80.00(65.00,102.25)** | **-0.549** | **0.583** |
| **Serum albumin, g/L** | **39.20(34.70,42.20)** | **34.60(28.38,40.35)** | **-2.579** | **0.010** | **40.50(35.20,43.90)** | **-1.583** | **0.113** |
| **Serum potassium, mmol/L** | **4.04(3.80,4.22)** | **4.04(3.76,4.24)** | **-0.007** | **0.995** | **4.03(3.80,4.28)** | **-0.644** | **0.520** |
| **CRP, mg/L** | **3.80(2.70,7.20)** | **3.50(2.38,5.90)** | **-1.135** | **0.256** | **3.80(2.58,5.73)** | **-0.518** | **0.605** |
| **Triglycerides, mmol/L** | **1.70(1.32,2.27)** | **1.68(1,14,2.42)** | **-0.670** | **0.497** | **1.53(1.04,2.48)** | **-1.224** | **0.221** |
| **Total cholesterol, mmol/L** | **5.28(4.6,6.04)** | **5.38(4.52,6.53)** | **-0.680** | **0.497** | **5.07(4.41,5.97)** | **-1.021** | **0.307** |
| **HDL-C, mmol/L** | **1.33(1.24,1.59)** | **1.34(1.09,1.63)** | **-0.663** | **0.507** | **1.28(1.08,1.57)** | **-1.711** | **0.087** |
| **LDL-C, mmol/L** | **3.05(2.58,3.37)** | **3.11(2.62,4.04)** | **-1.062** | **0.288** | **2.83(2.43,3.51)** | **-0.778** | **0.437** |
| **Platelet count, ×10^9^/L** | **214.00(184.00,237.00)** | **236.00(207.25,275.25)** | **-2.734** | **0.006** | **227.00(198.75,248.75)** | **-1.757** | **0.079** |
| **Hb, g/L** | **148.00(138.00,154.00)** | **128.00(118.00,139.00)** | **-5.179** | **<0.001** | **135.00(125.50,148.00)** | **-3.406** | **<0.001** |
| **Alkaline phosphatase, U/L** | **62.60(53.90,80.50)** | **66.00(57.23,77.15)** | **-0.731** | **0.465** | **68.60(58.45,82.18)** | **-1.552** | **0.121** |
| **Uric acid, µmol/L** | **337.00(302.00,384.00)** | **346.00(294.25,410.75)** | **-0.709** | **0.478** | **351.00(280.75,406.25)** | **-0.154** | **0.877** |
| **Serum phosphorus, mmol/L** | **1.21(1.05,1.34)** | **1.20(1.12,1.33)** | **-0.688** | **0.492** | **1.20(1.08,1.30)** | **-0.263** | **0.793** |
| **Serum sodium, mmol/L** | **139.50(138.80,141.20)** | **139.45(138.30,141.05)** | **-1.084** | **0.278** | **139.50(137.70,141.10)** | **-1.313** | **0.189** |
| **Serum chloride, mmol/L** | **105.70(103.90,107.30)** | **105.90(104.15,107.83)** | **-1.080** | **0.280** | **105.50(103.60,107.23)** | **-0.427** | **0.670** |
| **Serum bicarbonate, mmol/L** | **26.20(24.60,27.40)** | **26.25(24.08,27.80)** | **-0.483** | **0.629** | **25.30(23.90,27.20)** | **-1.879** | **0.060** |

Notes: ACEI, angiotensin converting enzyme inhibitors; ARB, angiotensin II receptor blockers; BMI, body mass index; SBP, systolic blood pressure; DBP, diastolic blood pressure; UACR, urinary albumin-to-creatinine ratio; eGFR, estimated glomerular filtration rate; Scr, serum creatinine; CRP, C-reactive protein; HDL-C, high‑density lipoprotein cholesterol; LDL‑C, low‑density lipoprotein cholesterol; Hb, hemoglobin.

**Supplementary Table S2.** Comparison of clinical efficacy at 3 months among the three groups before PSM

| **Response criteria** | **Combination**  **(n=59)** | **Finerenone**  **(n=110)** | **OR (95% CI)** | ***P*** | **Dapagliflozin**  **(n=158)** | **OR (95% CI)** | ***P*** |
| --- | --- | --- | --- | --- | --- | --- | --- |
| **Overall clinical remission rate (%)** | **38(64.41)** | **53(48.18)** | **1.95(1.02~3.73)** | **0.044** | **73(46.20)** | **2.11(1.14~3.91)** | **0.017** |
| **Complete remission (%)** | **13(22.03)** | **19(17.27)** |  |  | **27(17.09)** |  |  |
| **Partial remission (%)** | **25(42.37)** | **34(30.91)** |  |  | **46(29.11)** |  |  |
| **No response (%)** | **21(35.59)** | **57(51.82)** |  |  | **85(53.80)** |  |  |

**Supplementary Table S3.** Sensitivity analysis of clinical efficacy at 3 months among the three groups after PSM

| **Response criteria** | **Combination**  **(n=34)** | **Finerenone**  **(n=34)** | **OR (95% CI)** | ***P*** | **Combination**  **(n=39)** | **Dapagliflozin**  **(n=42)** | **OR (95% CI)** | ***P*** |
| --- | --- | --- | --- | --- | --- | --- | --- | --- |
| **Overall clinical remission rate (%)** | **24(70.59)** | **18** | **2.132(0.786-5.780)** | **0.134** | **26(66.67)** | **17(40.48)** | **2.941(1.188,7.299)** | **0.018** |
| **Complete remission (%)** | **9(26.47)** | **8** |  |  | **10(25.64)** | **7(16.67)** |  |  |
| **Partial remission (%)** | **15(44.12)** | **10** |  |  | **16(41.03)** | **10(23.81)** |  |  |
| **No response (%)** | **10(29.41)** | **16** |  |  | **13(33.33)** | **25(59.52)** |  |  |

**Supplementary Table S4.** Comparison of primary efficacy outcomes at 3 months among the three groups before PSM

| **Variable** | **Combination**  **(n=59)** | **Finerenone**  **(n=110)** | **t/Z** | ***P*** | **Dapagliflozin**  **(n=158)** | **t/Z** | ***P*** |
| --- | --- | --- | --- | --- | --- | --- | --- |
| **24‑hour urine volume, ml** | **1975.00(1500.00,2500.00)** | **1700.00(1250.00,2300.00)** | **-1.714** | **0.086** | **2100.00(1700.00,2700.00)** | **-1.595** | **0.111** |
| **UACR, mg/g** | **299.75(81.18,1117.53)** | **617.45(189.75,1472.58)** | **-2.080** | **0.038** | **304.90(138.60,673.98)** | **-0.014** | **0.989** |
| **24‑hour urinary protein, mg** | **659.50(312.75,1249.50)** | **1073.00(459.50,3055.00)** | **-2.462** | **0.014** | **702.00(380.00,1502.00)** | **-0.625** | **0.532** |
| **eGFR, mL·min^-1^·1.73 m^-2^** | **86.78±35.07** | **87.08±34.13** | **0.052** | **0.958** | **85.29±29.86** | **-0.302** | **0.763** |
| **Scr, µmol/L** | **82.00(65.50,112.50)** | **77.50(59.25,106.25)** | **-1.062** | **0.288** | **78.00(64.00,105.00)** | **-0.593** | **0.553** |
| **Δ SBP (%)** | **-3.10(-13.39,4.24)** | **-12.70(-20.57, -0.38)** | **-2.024** | **0.043** | **-3.57(-18.25,0.00)** | **-1.228** | **0.220** |
| **Δ DBP (%)** | **-2.70(-14.29,0.00)** | **-13.10(-23.21, -1.54)** | **-2.194** | **0.028** | **-7.41(-20.11,0.00)** | **-1.177** | **0.239** |
| **Δ UACR (%)** | **61.76(36.32,76.97)** | **57.62(28.11,71.48)** | **-1.320** | **0.187** | **48.46(32.50,69.35)** | **-1.880** | **0.060** |
| **Δ 24‑hour urinary protein (%)** | **55.73(35.66,73.05)** | **53.48(29.91,64.85)** | **-1.411** | **0.158** | **44.61(30.23,63.45)** | **-2.260** | **0.024** |
| **Δ eGFR (%)** | **-1.04(-7.06,4.26)** | **-1.6(-9.36,13.31)** | **-0.369** | **0.712** | **0.54(-9.53,8.40)** | **-0.470** | **0.638** |
| **Δ Scr (%)** | **1.09(-7.05,8.84)** | **2.08(-7.89,13.20)** | **-0.562** | **0.574** | **0.00(-7.89,8.56)** | **-0.179** | **0.858** |

Notes: Δ, percent change from baseline; other abbreviations as in Table 1. ^*^indicates comparison with baseline. Statistical significance is indicated as follows: ^*^*P* < 0.05, ^**^*P* < 0.01, ^***^*P* < 0.001.

**Supplementary Table S5.** Comparison of secondary efficacy outcomes at 3 months among the three groups before PSM

| **Variable** | **Combination**  **(n=59)** | **Finerenone**  **(n=110)** | **t/Z** | ***P*** | **Dapagliflozin**  **(n=158)** | **t/Z** | ***P*** |
| --- | --- | --- | --- | --- | --- | --- | --- |
| **SBP, mmHg** | **120.00(111.00,124.00)** | **121.00(110.00,132.00)** | **-0.866** | **0.386** | **110.00(105.00,125.00)** | **-1.029** | **0.304** |
| **DBP, mmHg** | **75.00(65.00,80.00)** | **72.00(68.50,81.00)** | **-0.057** | **0.955** | **75.00(68.50,80.00)** | **-0.062** | **0.950** |
| **CRP, mg/L** | **3.45(1.80,7.23)** | **3.50(2.05,5.10)** | **-0.380** | **0.704** | **3.30(1.93,5.10)** | **-0.438** | **0.661** |
| **Triglycerides, mmol/L** | **1.75(1.21,2.72)** | **1.61(1.06,2.78)** | **-0.681** | **0.496** | **1.53(1.04,2.29)** | **-1.771** | **0.076** |
| **Total cholesterol, mmol/L** | **5.40(4.53,6.23)** | **4.94(4.55,5.85)** | **-0.749** | **0.454** | **4.76(4.13,5.42)** | **-2.419** | **0.016** |
| **HDL-C, mmol/L** | **1.52(1.21,1.74)** | **1.32(1.19,1.60)** | **-1.519** | **0.129** | **1.30(1.08,1.64)** | **-2.402** | **0.016** |
| **LDL-C, mmol/L** | **2.86(2.34,3.58)** | **2.82(2.41,3.43)** | **-0.143** | **0.886** | **2.65(2.19,3.20)** | **-1.590** | **0.112** |
| **Platelet count, ×10^9^/L** | **219.98±69.05** | **232.29±70.34** | **0.921** | **0.359** | **227.69±60.53** | **0.669** | **0.505** |
| **Hb, g/L** | **146.45±21.01** | **129.07±17.62** | **-4.722** | **<0.001** | **140.31±18.93** | **-1.672** | **0.097** |
| **Alkaline phosphatase, U/L** | **65.20(54.30,82.60)** | **59.80(51.20,73.80)** | **-1.503** | **0.133** | **67.60(55.30,82.85)** | **-0.296** | **0.767** |
| **Serum albumin, g/L** | **40.30(36.93,43.10)** | **37.20(31.10,42.70)** | **-2.629** | **0.009** | **42.40(37.58,44.23)** | **-1.107** | **0.268** |
| **Uric acid, µmol/L** | **331.42±90.68** | **345.49±83.88** | **0.966** | **0.336** | **310.00(263.00,363.00)** | **-1.089** | **0.276** |
| **Serum phosphorus, mmol/L** | **1.19(1.08,1.34)** | **1.17(1.07,1.29)** | **-0.301** | **0.763** | **1.17(1.03,1.34)** | **-0.539** | **0.590** |
| **Serum potassium, mmol/L** | **4.25(3.92,4.47)** | **4.07(3.79,4.35)** | **-2.486** | **0.013** | **4.07(3.87,4.35)** | **-2.572** | **0.010** |
| **Serum sodium, mmol/L** | **139.20(137.50,140.10)** | **139.10(137.90,140.50)** | **-0.727** | **0.467** | **139.10(137.60,140.60)** | **-0.495** | **0.621** |
| **Serum chloride, mmol/L** | **105.00(103.35,106.55)** | **105.30(103.30,107.10)** | **-0.674** | **0.500** | **105.10(103.80,107.15)** | **-0.530** | **0.596** |
| **Serum bicarbonate, mmol/L** | **25.35(23.25,28.60)** | **26.30(24.50,28.40)** | **-0.933** | **0.351** | **25.40(23.73,27.60)** | **-0.552** | **0.581** |

Notes: abbreviations as in Supplementary Table S1.

**Supplementary Table S6.** Comparison of other secondary efficacy outcomes at 3 months among the three groups after PSM

| **Variable** | **Combination**  **(n=41)** | **Finerenone**  **(n=41)** | **t/Z** | ***P*** | **Combination**  **(n=46)** | **Dapagliflozin**  **(n=46)** | **t/Z** | ***P*** |
| --- | --- | --- | --- | --- | --- | --- | --- | --- |
| **CRP, mg/L** | **5.12±5.50** | **4.14±3.03** | **-0.876** | **0.385** | **3.45(1.68,7.28)** | **3.30(2.10,5.20)** | **-0.070** | **0.944** |
| **Triglycerides, mmol/L** | **1.71(1.14,2.39)** | **1.61(1.05,2.78)** | **-0.188** | **0.851** | **1.81(1.48,2.54)** | **1.81(1.15,2.18)** | **-0.851** | **0.395** |
| **Total cholesterol, mmol/L** | **5.11±1.16** | **5.46±1.51** | **1.043** | **0.301** | **5.40(4.53,6.16)** | **5.11(3.89,6.15)** | **-0.328** | **0.743** |
| **HDL-C, mmol/L** | **1.56(1.23,1.82)** | **1.37(1.18,1.61)** | **-1.390** | **0.165** | **1.53±0.40** | **1.41±0.40** | **-1.361** | **0.178** |
| **LDL-C, mmol/L** | **2.74±0.74** | **3.06±0.89** | **1.606** | **0.113** | **2.73(2.25,3.51)** | **2.80(2.15,3.49)** | **-0.035** | **0.972** |
| **Platelet count, ×10^9^/L** | **216.73±64.65** | **231.06±74.16** | **0.803** | **0.425** | **219.37±72.90** | **214.97±59.25** | **-0.268** | **0.789** |
| **Hb, g/L** | **147.04±21.21** | **128.87±20.20** | **-3.368** | **0.001** | **143.43±23.09** | **136.09±19.86** | **-1.356** | **0.180** |
| **Alkaline phosphatase, U/L** | **70.30(55.50,82.60)** | **60.75(52.13,72.88)** | **-1.136** | **0.256** | **71.07±23.49** | **68.26±21.68** | **-0.535** | **0.594** |
| **Uric acid, µmol/L** | **331.43±93.24** | **348.85±88.50** | **0.841** | **0.403** | **330.61±95.22** | **347.37±86.06** | **0.862** | **0.391** |
| **Serum phosphorus, mmol/L** | **1.21±0.19** | **1.18±0.15** | **-0.613** | **0.542** | **1.19±0.19** | **1.19±0.17** | **-0.200** | **0.842** |
| **Serum potassium, mmol/L** | **4.36±0.45 ^***^** | **4.19±0.54** | **-1.599** | **0.114** | **4.32±0.43 ^**^** | **4.14±0.37** | **-2.155** | **0.034** |
| **Serum sodium, mmol/L** | **139.10(137.40,139.95) ^**^** | **138.85(137.90,141.03)** | **-1.030** | **0.303** | **139.09±1.94** | **139.42±1.95** | **0.805** | **0.423** |
| **Serum chloride, mmol/L** | **104.97±2.76** | **105.08±3.12** | **0.167** | **0.867** | **105.32±2.72** | **105.42±3.09** | **0.161** | **0.873** |
| **Serum bicarbonate, mmol/L** | **25.67±3.52** | **26.46±2.57** | **1.102** | **0.275** | **26.21±3.19** | **26.00±2.80** | **-0.322** | **0.748** |

Notes: abbreviations as in Supplementary Table S1. ^*^indicates comparison with baseline. Statistical significance is indicated as follows: ^*^*P* < 0.05, ^**^*P* < 0.01, ^***^*P* < 0.001**.**

**Supplementary Table S7.** Comparison of safety outcomes at 3 months among the three groups before PSM

| **Adverse reaction** | **Combination**  **(n=59)** | **Finerenone**  **(n=110)** | ***P*** | **Dapagliflozin**  **(n=158)** | ***P*** |
| --- | --- | --- | --- | --- | --- |
| **Incidence of adverse reactions (%)** | **4(6.78)** | **4(3.64)** | **0.452** | **11(6.96)** | **1.000** |
| **Hyperkalemia (%)** | **4(6.78)** | **4(3.64)** |  | **2(1.27)** |  |
| **Urinary tract infection (%)** | **0** | **0** |  | **8(5.06)** |  |
| **Hypoglycemia (%)** | **0** | **0** |  | **1(0.63)** |  |

**Supplementary Table S8.** Comparison of treatment costs (RMB) among the three groups after PSM

| **Group** | **Medication cost (RMB)** | **Examination cost (RMB)** | **Average treatment cost per patient (RMB)** |
| --- | --- | --- | --- |
| **Combination**  **(n=59)** | **1025.10** | **655.12** | **1680.22** |
| **Finerenone**  **(n=110)** | **632.70** | **655.12** | **1287.82** |
| **Dapagliflozin**  **(n=158)** | **392.40** | **655.12** | **1047.52** |

**Supplementary Table S9.** Probabilistic sensitivity analysis results for combination therapy versus monotherapy (1,000 Monte Carlo simulations)

| **Comparison** | **ICER median (RMB/%)** | **95% uncertainty interval** | **Probability of ΔE > 0 (%)** | **Probability of ΔC > 0 and ΔE > 0 (%)** |
| --- | --- | --- | --- | --- |
| **Combination vs. Finerenone** | **23.26** | **-32.05~186.07** | **94.80** | **79.70** |
| **Combination vs. Dapagliflozin** | **20.82** | **-3.71~74.53** | **99.80** | **94.60** |

Notes: ICER, incremental cost-effectiveness ratio; ΔE, incremental effect; ΔC, incremental cost. ICER is expressed as RMB per 1% increase in remission rate. PSA was performed using Monte Carlo simulation with 1,000 iterations.

**Supplementary Table S10.** Wald type III tests of effects from generalized estimating equation model for clinical remission (3–6 months follow-up)

| **Effect (3‑6 months)** | **Wald‑χ²** | ***P*** |
| --- | --- | --- |
| **Time effect** | **8.597** | **0.003** |
| **Group effect** | **2.055** | **0.358** |
| **Group × Time interaction** | **1.043** | **0.594** |

**Supplementary Table S11.** Clinical remission rates at different follow-up time points and GEE model effect tests (3–24 months)

| **Variable** | **Combination** | **Finerenone** | **Dapagliflozin** | **Time effect** | | **Group effect** | | **Group × Time interaction** | |
| --- | --- | --- | --- | --- | --- | --- | --- | --- | --- |
|  |  |  |  | **Wald-χ²** | ***P*** | **Wald-χ²** | ***P*** | **Wald-χ²** | ***P*** |
| **3 months** | **38(64.41)**  **(n=59)** | **53(48.18)**  **(n=110)** | **73(46.20)**  **(n=158)** | **68.004** | **<0.001** | **4.354** | **0.113** | **1.809** | **0.405** |
| **6 months** | **15(75.00)**  **(n=20)** | **35(74.47)**  **(n=47)** | **56(61.54)**  **(n=91)** |  |  |  |  |  |  |
| **9 months** | **10(76.92)**  **(n=13)** | **22(84.62)**  **(n=26)** | **45(75.00)**  **(n=60)** |  |  |  |  |  |  |
| **12 months** | **8(100.00)**  **(n=8)** | **24(88.89)**  **(n=27)** | **41(82.00)**  **(n=50)** |  |  |  |  |  |  |
| **15 months** | **4(100.00)**  **(n=4)** | **12(85.71)**  **(n=14)** | **31(91.18)**  **(n=34)** |  |  |  |  |  |  |
| **18 months** | **1(100.00)**  **(n=1)** | **10(100.00)**  **(n=10)** | **22(95.65)**  **(n=23)** |  |  |  |  |  |  |
| **21 months** | **2(100.00)**  **(n=2)** | **4(100.00)**  **(n=4)** | **17(80.95)**  **(n=21)** |  |  |  |  |  |  |
| **24 months** | **2(100.00)**  **(n=2)** | **2(100.00)**  **(n=2)** | **10(100.00)**  **(n=10)** |  |  |  |  |  |  |

**Supplementary Table S12.** Wald type III tests of effects from generalized estimating equation model for 24‑hour urinary protein (3–6 months follow-up)

| **Effect (3‑6 months)** | **Wald‑χ²** | ***P*** |
| --- | --- | --- |
| **Time effect** | **17.101** | **0.001** |
| **Group effect** | **10.175** | **0.006** |
| **Group × Time interaction** | **4.681** | **0.096** |

**Supplementary Table S13.** Median 24‑hour urinary protein at different follow-up time points and GEE model effect tests (3–24 months)

| **Variable** | **Combination** | **Finerenone** | **Dapagliflozin** | **Time effect** | | **Group effect** | | **Group × Time interaction** | |
| --- | --- | --- | --- | --- | --- | --- | --- | --- | --- |
|  |  |  |  | **Wald-χ²** | ***P*** | **Wald-χ²** | ***P*** | **Wald-χ²** | ***P*** |
| **Baseline** | **2339.00(1030.00,4296.00)**  **(n=59)** | **2696.00(939.00,5038.75)**  **(n=110)** | **1504.00(801.75,2725.25)**  **(n=158)** | **44.416** | ***0.001*** | **3.243** | ***0.198*** | ***3.821*** | ***0.148*** |
| **3 months** | **664.00(313.50,1307.50) ^***^**  **(n=48)** | **1073.00(459.50,3055.00) ^***^**  **(n=96)** | **702.00(380.00,1502.00) ^***^**  **(n=145)** |  |  |  |  |  |  |
| **6 months** | **572.00(207.50,949.00)**  **(n=17)** | **661.00(255.00,1433.00) ^**^**  **(n=41)** | **530.00(325.00,893.00) ^**^**  **(n=83)** |  |  |  |  |  |  |
| **9 months** | **492.00(296.25,628.00)**  **(n=12)** | **376.00(163.00,661.00)**  **(n=23)** | **399.00(283.00,707.50)**  **(n=57)** |  |  |  |  |  |  |
| **12 months** | **328.00(169.75,426.75)**  **(n=8)** | **355.00(181.75,754.25)**  **(n=24)** | **383.50(216.75,587.75)**  **(n=46)** |  |  |  |  |  |  |
| **15 months** | **267.00(153.00,820.00)**  **(n=4)** | **337.00(153.00,820.00)**  **(n=13)** | **345.00(295.00,447.00)**  **(n=31)** |  |  |  |  |  |  |
| **18 months** | **274.00**  **(n=1)** | **346.50(182.25,525.25)**  **(n=9)** | **264.00(174.50,416.00)**  **(n=21)** |  |  |  |  |  |  |
| **21 months** | **223.50(140.00,307.00)**  **(n=2)** | **264.50(193.75,1269.00)**  **(n=4)** | **305.00(161.50,557.50)**  **(n=20)** |  |  |  |  |  |  |
| **24 months** | **167**  **(n=1)** | **152.50(105.00,200.00)**  **(n=2)** | **314.00(203.00,482.00)**  **(n=9)** |  |  |  |  |  |  |

**Supplementary Table S14.** Wald type III tests of effects from generalized estimating equation model for the rate of reduction in 24-hour urinary protein (3–6 months follow-up) in the full unmatched cohort.

| **Effect (3‑6 months)** | **Wald‑χ²** | ***P*** |
| --- | --- | --- |
| **Time effect** | **18.683** | **0.001** |
| **Group effect** | **4.631** | **0.099** |
| **Group × Time interaction** | **2.608** | **0.271** |

**Supplementary Table S15.** Median rate of reduction in 24-hour urinary protein at different follow-up time points and GEE model effect tests (3–24 months) in the full unmatched cohort.

| **Variable** | **Combination** | **Finerenone** | **Dapagliflozin** | **Time effect** | | **Group effect** | | **Group × Time interaction** | |
| --- | --- | --- | --- | --- | --- | --- | --- | --- | --- |
|  |  |  |  | **Wald-χ²** | ***P*** | **Wald-χ²** | ***P*** | **Wald-χ²** | ***P*** |
| **3 months** | **55.73(35.66,73.05)**  **(n=48)** | **53.48(29.91,64.85)**  **(n=96)** | **44.61(30.23,63.45)**  **(n=145)** | **147.054** | ***0.001*** | **4.902** | ***0.086*** | ***2.364*** | ***0.307*** |
| **6 months** | **65.93(48.03,77.37)**  **(n=17)** | **60.04(51.06,74.10) ^**^**  **(n=40)** | **58.57(41.41,74.28) ^***^**  **(n=81)** |  |  |  |  |  |  |
| **9 months** | **57.66(44.57,89.67)**  **(n=12)** | **77.91(47.37,89.12)**  **(n=22)** | **61.08(48.90,83.14)**  **(n=56)** |  |  |  |  |  |  |
| **12 months** | **83.51(65.43,90.05)**  **(n=8)** | **85.00(54.54,93.49)**  **(n=23)** | **69.25(54.58,83.41)**  **(n=45)** |  |  |  |  |  |  |
| **15 months** | **84.76(74.42,91.60)**  **(n=4)** | **88.10(74.59,94.38)**  **(n=13)** | **77.72(57.34,91.50)**  **(n=30)** |  |  |  |  |  |  |
| **18 months** | **90.66**  **(n=1)** | **82.44(62.59,90.69)**  **(n=9)** | **78.35(57.49,84.43)**  **(n=21)** |  |  |  |  |  |  |
| **21 months** | **86.30(83.05,89.54)**  **(n=2)** | **80.13(72.66,84.91)**  **(n=3)** | **77.63(51.60,91.84)**  **(n=20)** |  |  |  |  |  |  |
| **24 months** | **94.31**  **(n=1)** | **71.09(48.78,93.40)**  **(n=2)** | **80.50(62.23,95.75)**  **(n=9)** |  |  |  |  |  |  |

**Supplementary Table S16.** Wald type III tests of effects from generalized estimating equation model for estimated glomerular filtration rate (eGFR) (3–6 months follow-up) in the full unmatched cohort.

| **Effect (3‑6 months)** | **Wald‑χ²** | ***P*** |
| --- | --- | --- |
| **Time effect** | **0.008** | **0.928** |
| **Group effect** | **1.836** | **0.399** |
| **Group × Time interaction** | **2.369** | **0.306** |

**Supplementary Table S17.** Mean eGFR at different follow-up time points and GEE model effect tests (3–24 months) in the full cohort.

| **Variable** | **Combination** | **Finerenone** | **Dapagliflozin** | **Time effect** | | **Group effect** | | **Group × Time interaction** | |
| --- | --- | --- | --- | --- | --- | --- | --- | --- | --- |
|  |  |  |  | **Wald-χ²** | **P** | **Wald-χ²** | **P** | **Wald-χ²** | **P** |
| **Baseline** | **86.74±31.53**  **(n=59)** | **91.02±35.50**  **(n=110)** | **86.06±28.55**  **(n=158)** | **0.043** | **0.836** | **1.290** | **0.525** | **1.181** | **0.554** |
| **3 months** | **87.10±35.32**  **(n=54)** | **87.08±34.13**  **(n=107)** | **85.29±29.86**  **(n=152)** |  |  |  |  |  |  |
| **6 months** | **91.14±28.21**  **(n=16)** | **95.21±28.18**  **(n=43)** | **85.74±31.13**  **(n=89)** |  |  |  |  |  |  |
| **9 months** | **96.11±30.57**  **(n=10)** | **98.88±27.22**  **(n=24)** | **84.15±25.00**  **(n=57)** |  |  |  |  |  |  |
| **12 months** | **98.87±25.17**  **(n=7)** | **88.60±25.45**  **(n=26)** | **83.50±26.99**  **(n=48)** |  |  |  |  |  |  |
| **15 months** | **103.55±14.70**  **(n=4)** | **89.76±24.73**  **(n=13)** | **77.48±25.29**  **(n=31)** |  |  |  |  |  |  |
| **18 months** | **77**  **(n=1)** | **84.91±22.99**  **(n=10)** | **80.79±27.88**  **(n=23)** |  |  |  |  |  |  |
| **21 months** | **131.90±58.83**  **(n=2)**  **=2** | **92.95±25.58**  **(n=4)** | **90.72±23.86**  **(n=19)** |  |  |  |  |  |  |
| **24 months** | **69.55±31.18**  **(n=2)** | **96.15±5.44**  **(n=2)** | **91.30±22.60**  **(n=10)** |  |  |  |  |  |  |

**Supplementary Table S18.** Comparison of UACR at different time points among the three groups in the full unmatched cohort.

| **Time point** | **Combination** | **Finerenone** | **Dapagliflozin** |
| --- | --- | --- | --- |
| **Baseline** | **1183.80(390.40,3631.80)**  **(n=59)** | **1536.80(499.08,3013.43)**  **(n=110)** | **774.30(328.70,1346.50)**  **(n=158)** |
| **3 months** | **299.75(81.18,1117.53)**  **(n=56)** | **617.45(189.75,1472.58)**  **(n=104)** | **304.90(138.60,673.98)**  **(n=136)** |
| **6 months** | **239.10(22.23,442.88)**  **(n=16)** | **426.50(130.13,900.30)**  **(n=42)** | **219.90(80.90,431.80)**  **(n=73)** |
| **9 months** | **214.10(38.10,606.40)**  **(n=11)** | **376.00(163.00,661.00)**  **(n=25)** | **142.30(89.35,344.40)**  **(n=48)** |
| **12 months** | **59.80(22.75,280.00)**  **(n=5)** | **166.60(42.68,326.00)**  **(n=26)** | **115.15(68.75,309.95)**  **(n=42)** |
| **15 months** | **28.40(26.15,171.65)**  **(n=3)** | **222.95(64.73,757.60)**  **(n=14)** | **132.05(78.18,243.98)**  **(n=30)** |
| **18 months** | **30.60**  **(n=1)** | **217.05(68.60,661.48)**  **(n=10)** | **98.80(43.25,236.35)**  **(n=21)** |
| **21 months** | **23.75(6.40,41.10)**  **(n=2)** | **92.20(59.70,731.83)**  **(n=4)** | **77.80(32.90,188.70)**  **(n=19)** |
| **24 months** | **25.55(22.20,28.90)**  **(n=2)** | **30.10(26.70,33.50)**  **(n=2)** | **66.20(20.60,254.60)**  **(n=9)** |

**Supplementary Table S19.** Comparison of the rate of reduction in UACR at different time points among the three groups in the full unmatched cohort.

| **Time point** | **Combination** | **Finerenone** | **Dapagliflozin** |
| --- | --- | --- | --- |
| **3 months** | **61.76(36.32,76.97)**  **(n=56)** | **57.62(28.11,71.48)**  **(n=104)** | **48.46(32.50,69.35)**  **(n=136)** |
| **6 months** | **64.86(37.89,90.87)**  **(n=16)** | **68.35(43.50,80.51)**  **(n=42)** | **57.91(36.90,78.52)**  **(n=67)** |
| **9 months** | **68.87(37.82,94.94)**  **(n=11)** | **79.34(62.39,91.18)**  **(n=25)** | **58.76(26.99,85.37)**  **(n=42)** |
| **12 months** | **91.10(70.26,95.97)**  **(n=5)** | **89.57(69.20,95.24)**  **(n=26)** | **69.20(55.19,87.38)**  **(n=35)** |
| **15 months** | **91.31(86.22,94.70)**  **(n=3)** | **90.02(70.66,96.13)**  **(n=14)** | **84.46(53.54,94.98)**  **(n=27)** |
| **18 months** | **97.56**  **(n=1)** | **90.03(56.22,95.51)**  **(n=10)** | **75.69(51.98,95.42)**  **(n=17)** |
| **21 months** | **97.61(96.72,98.50)**  **(n=2)** | **94.07(47.06,96.36)**  **(n=4)** | **88.97(64.42,97.36)**  **(n=16)** |
| **24 months** | **98.71(98.23,99.18)**  **(n=2)** | **74.89(51.54,98.24)**  **(n=2)** | **87.58(69.09,98.47)**  **(n=8)** |

**Supplementary Table S20.** Comparison of safety at different time points among the three groups in the full unmatched cohort.

| **Time point** | **Combination** | **Finerenone** | **Dapagliflozin** |
| --- | --- | --- | --- |
| **3 months** | **4(6.78)**  **(n=59)** | **4(3.64)**  **(n=110)** | **11(6.96)**  **(n=158)** |
| **6 months** | **1(5.00)**  **(n=20)** | **1(2.13)**  **(n=47)** | **6(6.59)**  **(n=91)** |
| **9 months** | **1(7.69)**  **(n=13)** | **0(0.00)**  **(n=26)** | **4(6.67)**  **(n=60)** |
| **12 months** | **1(12.50)**  **(n=8)** | **0(0.00)**  **(n=27)** | **3(6.00)**  **(n=50)** |
| **15 months** | **0(0.00)**  **(n=4)** | **0(0.00)**  **(n=14)** | **3(8.82)**  **(n=34)** |
| **18 months** | **0(0.00)**  **(n=1)** | **0(0.00)**  **(n=10)** | **1(4.35)**  **(n=23)** |
| **21 months** | **0(0.00)**  **(n=2)** | **0(0.00)**  **(n=4)** | **0(0.00)**  **(n=21)** |
| **24 months** | **0(0.00)**  **(n=2)** | **0(0.00)**  **(n=2)** | **0(0.00)**  **(n=10)** |

**Supplementary Table S21.** Membranous nephropathy subgroups: Comparison of baseline characteristics at 3 months among the three groups after PSM

| **Variable** | **Combination**  **(n=21)**  **n** | **Finerenone**  **(n=21)** | **t/Z/X^2^** | ***P*** | **Combination**  **(n=30)** | **Dapagliflozin**  **(n=20)** | **t/Z/X^2^** | ***P*** |
| --- | --- | --- | --- | --- | --- | --- | --- | --- |
| **Age, years** | **53.24±16.79** | **55.71±14.24** | **0.515** | **0.609** | **48.00**(**34.75,66.25**) | **57.00**(**50.75,60.00**) | **-0.714** | **0.475** |
| **Sex, male, n (%)** | **12(57.14)** | **14(66.67)** | **0.404** | **0.525** | **20(66.67)** | **14(70.00)** | **0.061** | **0.804** |
| **Weight, kg** | **68.68±14.54** | **69.60±11.23** | **0.229** | **0.820** | **70.00(60.43,78.58)** | **66.00**(**61.25,68.38**) | **-1.546** | **0.122** |
| **BMI, kg/m^2^** | **23.88(23.33,26.09)** | **25.65(22.74,28.14)** | **-0.805** | **0.421** | **24.39**(**23.46,27.44**) | **24.63(21.89,25.82)** | **-0.505** | **0.614** |
| **SBP, mmHg** | **122.00(113.50,125.00)** | **135.00(116.50,140.00)** | **-1.263** | **0.207** | **122.00(112.00,124.00)** | **124.00(118.25,132.25)** | **-0.893** | **0.372** |
| **DBP, mmHg** | **77.62±10.03** | **81.81±6.50** | **1.607** | **0.116** | **75.90±10.84** | **79.00±12.47** | **0.933** | **0.356** |
| **Hyperlipidemia, n (%)** | **15(71.43)** | **10(47.62)** | **2.471** | **0.116** | **21(70.00)** | **12(60.00)** | **0.535** | **0.465** |
| **Medication** |  |  |  |  |  |  |  |  |
| **ACEI, n (%)** | **0(0.00)** | **0(0.00)** |  |  | **0(0.00)** | **2(10.00)** |  |  |
| **ARB, n (%)** | **9(42.86)** | **5(23.81)** |  |  | **15(50.00)** | **9(45.00)** |  |  |
| **Calcium channel blocker, n (%)** | **8(38.10)** | **4(19.05)** |  |  | **11(36.67)** | **2(10.00)** |  |  |
| **Diuretic, n (%)** | **0(0.00)** | **2(9.52)** |  |  | **1(3.33)** | **0(0.00)** |  |  |
| **β‑blocker, n (%)** | **2(9.52)** | **1(4.76)** |  |  | **3(10.00)** | **1(5.00)** |  |  |
| **α‑blocker, n (%)** | **0(0.00)** | **0(0.00)** |  |  | **0(0.00)** | **0(0.00)** |  |  |
| **Centrally acting antihypertensive, n (%)** | **0(0.00)** | **0(0.00)** |  |  | **0(0.00)** | **0(0.00)** |  |  |
| **Untreated (%)** | **0(0.00)** | **0(0.00)** |  |  | **0(0.00)** | **0(0.00)** |  |  |
| **24‑hour urine volume, ml** | **2300.00(1950.00,2550.00)** | **1900.00(1325.00,2200.00)** | **-2.055** | **0.040** | **2343.00±598.44** | **1990.00±711.12** | **-1.895** | **0.063** |
| **UACR, mg/g** | **1575.70(730.65,4936.05)** | **2804.80(1739.15,6060.15)** | **-1.421** | **0.155** | **1510.25(597.53,4594.60)** | **1819.15(850.85,5415.30)** | **-1.030** | **0.303** |
| **24‑hour urinary protein, mg** | **2935.00(1161.50,6033.50)** | **4249.00(2747.00,8003.00)** | **-1.296** | **0.195** | **2910.50(1061.25,7647.00)** | **5025.00(2116.25,10223.25)** | **-1.416** | **0.157** |
| **eGFR, mL·min^-1^·1.73 m^-2^** | **87.40(68.30,102.35)** | **87.60(62.80,110.70)** | **-0.440** | **0.660** | **93.85(73.73,102.55)** | **94.30(76.05,106.60)** | **-0.416** | **0.677** |
| **Scr, µmol/L** | **77.00(68.00,108.00)** | **79.00(64.00,110.00)** | **-0.277** | **0.782** | **79.50(69.50,94.25)** | **74.00(62.50,94.25)** | **-1.298** | **0.194** |
| **CRP, mg/L** | **4.70(2.85,8.40)** | **4.20(3.30,6.85)** | **-0.088** | **0.930** | **3.80(2.50,7.75)** | **3.65(2.10,4.83)** | **-1.031** | **0.302** |
| **Triglycerides, mmol/L** | **1.64(1.25,2.10)** | **2.08(1.29,2.81)** | **-1.346** | **0.178** | **1.70(1.34,2.32)** | **2.32(1.61,3.02)** | **-1.853** | **0.064** |
| **Total cholesterol, mmol/L** | **5.28(4.55,6.31)** | **5.98(5.38,7.47)** | **-1.913** | **0.056** | **5.28(4.60,6.23)** | **5.07(4.58,6.63)** | **-0.050** | **0.960** |
| **HDL-C, mmol/L** | **1.33(1.25,1.70)** | **1.38(1.07,1.65)** | **-0.252** | **0.801** | **1.33(1.24,1.63)** | **1.28(1.14,1.55)** | **-1.279** | **0.201** |
| **LDL-C, mmol/L** | **3.05(2.59,3.78)** | **3.74(2.94,4.40)** | **-1.875** | **0.061** | **3.05(2.58,3.71)** | **2.88(2.48,3.92)** | **-0.168** | **0.866** |
| **Platelet count, ×10^9^/L** | **211.24±48.42** | **246.00±38.92** | **2.564** | **0.014** | **214.00(200.50,246.50)** | **227.00(191.25,256.25)** | **-0.806** | **0.420** |
| **Alkaline phosphatase, U/L** | **68.13±19.90** | **72.94±19.16** | **0.797** | **0.430** | **68.34±18.64** | **76.60±16.02** | **1.622** | **0.111** |
| **Serum albumin, g/L** | **36.18±7.07** | **30.55±6.58** | **-2.668** | **0.011** | **36.47±7.17** | **33.68±6.96** | **-1.363** | **0.179** |
| **Uric acid, µmol/L** | **339.90±60.66** | **374.33±103.75** | **1.313** | **0.199** | **342.67±60.09** | **358.65±81.94** | **0.796** | **0.430** |
| **Serum phosphorus, mmol/L** | **1.17±0.20** | **1.23±0.16** | **1.038** | **0.306** | **1.19±0.19** | **1.21±0.16** | **0.256** | **0.799** |
| **Serum potassium, mmol/L** | **4.04±0.34** | **4.02±0.38** | **-0.193** | **0.848** | **4.03±0.36** | **4.07±0.61** | **0.283** | **0.779** |
| **Serum sodium, mmol/L** | **140.96±2.09** | **139.86±2.33** | **-1.612** | **0.115** | **139.70(138.73,141.45)** | **139.50(137.93,141.23)** | **-1.169** | **0.242** |
| **Serum chloride, mmol/L** | **105.93±2.24** | **107.27±2.68** | **1.760** | **0.086** | **105.75(103.85,107.33)** | **104.60(103.38,106.25)** | **-1.020** | **0.308** |
| **Serum bicarbonate, mmol/L** | **27.29±2.91** | **25.97±3.01** | **-1.448** | **0.155** | **26.60(25.23,28.93)** | **27.25(24.35,30.45)** | **-0.307** | **0.759** |

Notes: abbreviations as in Supplementary Table S1.

**Supplementary Table S22.** Membranous nephropathy subgroups: Comparison of secondary efficacy outcomes at 3 months among the three groups after PSM

| **Variable** | **Combination**  **(n=21)**  **n** | **Finerenone**  **(n=21)** | **t/Z** | ***P*** | **Combination**  **(n=30)** | **Dapagliflozin**  **(n=20)** | **t/Z** | ***P*** |
| --- | --- | --- | --- | --- | --- | --- | --- | --- |
| **SBP, mmHg** | **119.00(107.50,120.00)** | **117.00(110.75,131.75)** | **-0.849** | **0.396** | **118.00(109.50,120.00)** | **110.00(95.00,126.25)** | **-0.439** | **0.661** |
| **DBP, mmHg** | **70.10±12.03** | **73.00±5.81** | **0.671** | **0.514** | **75.00(62.50,80.00)** | **71.00(54.50,80.00)** | **-1.056** | **0.291** |
| **CRP, mg/L** | **3.15(1.28,4.83)** | **3.10(2.05,6.15)** | **-0.337** | **0.736** | **2.85(1.28,7.23)** | **3.00(1.80,4.15)** | **-0.113** | **0.910** |
| **Triglycerides, mmol/L** | **1.71(1.21,1.96)** | **1.64(1.26,3.14)** | **-0.860** | **0.390** | **1.78(1.49,2.47)** | **1.93(1.57,2.20)** | **-0.602** | **0.547** |
| **Total cholesterol, mmol/L** | **5.38±0.95** | **5.76±1.55** | **0.828** | **0.413** | **5.47(4.72,6.22)** | **4.86(4.15,6.04)** | **-1.076** | **0.282** |
| **HDL-C, mmol/L** | **1.60(1.33,2.12)** | **1.43(1.27,1.87)** | **-1.011** | **0.312** | **1.60±0.43** | **1.37±0.36** | **-1.788** | **0.082** |
| **LDL-C, mmol/L** | **2.98±0.61** | **3.17±0.89** | **0.738** | **0.465** | **3.16(2.46,3.71)** | **2.73(2.20,3.48)** | **-0.725** | **0.468** |
| **Platelet count, ×10^9^/L** | **211.38±68.41** | **256.61±65.42** | **1.864** | **0.073** | **233.53±68.24** | **210.62±63.08** | **-0.961** | **0.344** |
| **Alkaline phosphatase, U/L** | **76.81±27.30** | **71.32±19.01** | **-0.713** | **0.481** | **75.71±24.06** | **70.51±19.54** | **-0.724** | **0.473** |
| **Serum albumin, g/L** | **38.92±5.26** | **33.92±6.59** | **-2.536** | **0.016** | **38.72±5.83** | **35.22±7.07** | **-1.777** | **0.083** |
| **Uric acid, µmol/L** | **340.40±105.69** | **369.90±98.21** | **0.914** | **0.366** | **333.04±96.69** | **356.90±95.82** | **0.846** | **0.402** |
| **Serum phosphorus, mmol/L** | **1.26±0.17** | **1.18±0.15** | **-1.552** | **0.129** | **1.23±0.19** | **1.25±0.16** | **0.475** | **0.637** |
| **Serum potassium, mmol/L** | **4.49±0.46** | **4.22±0.55** | **-1.712** | **0.095** | **4.35±0.46** | **4.25±0.34** | **-0.893** | **0.376** |
| **Serum sodium, mmol/L** | **139.01±2.12** | **139.13±2.13** | **0.186** | **0.853** | **139.24±1.90** | **139.84±1.61** | **1.152** | **0.255** |
| **Serum chloride, mmol/L** | **104.95±3.19** | **106.00±3.14** | **1.061** | **0.295** | **105.07±2.80** | **106.26±2.58** | **1.505** | **0.139** |
| **Serum bicarbonate, mmol/L** | **26.57±3.08** | **26.17±2.52** | **-0.454** | **0.652** | **26.69±3.07** | **26.55±2.23** | **-0.183** | **0.855** |

Notes: abbreviations as in Supplementary Table S1.

**Supplementary Table S23.** Membranous nephropathy subgroups: Comparison of safety at 3 months among the three groups after PSM

| **Adverse reaction** | **Combination**  **(n=21)**  **n** | **Finerenone**  **(n=21)** | ***P*** | **Combination**  **(n=30)** | **Dapagliflozin**  **(n=20)** | ***P*** |
| --- | --- | --- | --- | --- | --- | --- |
| **Incidence of adverse reactions (%)** | **3(14.29)** | **3(14.29)** | **1.000** | **3(10.00)** | **1(5.00)** | **0.641** |
| **Hyperkalemia (%)** | **3(14.29)** | **3(14.29)** |  | **3(10.00)** | **0(0.00)** |  |
| **Urinary tract infection (%)** | **0(0.00)** | **0(0.00)** |  | **0(0.00)** | **1(5.00)** |  |

**Supplementary Table S24.** Hypertension subgroups: Comparison of baseline characteristics at 3 months among the three groups after PSM

| **Variable** | **Combination**  **(n=27)**  **n** | **Finerenone**  **(n=24)** | **t/Z/X^2^** | ***P*** | **Combination**  **(n=31)** | **Dapagliflozin**  **(n=24)** | **t/Z/X^2^** | ***P*** |
| --- | --- | --- | --- | --- | --- | --- | --- | --- |
| **Age, years** | **55.15±18.55** | **50.17±3.32** | **-1.001** | **0.322** | **52.71±17.99** | **53.75±13.74** | **0.243** | **0.809** |
| **Sex, male, n (%)** | **16(59.26)** | **17(70.83)** | **0.745** | **0.388** | **20(64.52)** | **12(50.00)** | **1.172** | **0.279** |
| **Weight, kg** | **67.58±13.22** | **70.91±11.98** | **0.939** | **0.353** | **70.00(58.90,78.00)** | **64.50(61.00,71.13)** | **-0.569** | **0.569** |
| **BMI, kg/m^2^** | **24.15±3.32** | **24.89±3.63** | **0.755** | **0.454** | **24.39(22.86,27.27)** | **24.32(23.68,26.21)** | **-0.008** | **0.993** |
| **SBP, mmHg** | **122.00(120.00,134.00)** | **135.00(118.75,138.00)** | **-1.154** | **0.248** | **122.00(115.00,132.00)** | **130.00(118.50,141.75)** | **-1.420** | **0.156** |
| **DBP, mmHg** | **76.00(75.00,89.00)** | **84.00(76.00,89.75)** | **-1.079** | **0.281** | **75.00(71.00,85.00)** | **80.00(71.25,88.75)** | **-1.084** | **0.278** |
| **Histological type** |  |  | **1.196** | **0.780** |  |  | **2.191** | **0.586** |
| **Membranous nephropathy, n (%)** | **15(55.56)** | **10(41.67)** |  |  | **21(67.74)** | **12(50.00)** |  |  |
| **IgA nephropathy, n (%)** | **5(18.52)** | **6(25.00)** |  |  | **7(22.58)** | **8(33.33)** |  |  |
| **Primary glomerular disease, n (%)** | **2(7.41)** | **2(8.33)** |  |  | **1(4.17)** | **1(4.17)** |  |  |
| **Secondary / other nephropathy, n (%)** | **5(18.52)** | **6(25.00)** |  |  | **2(6.45)** | **3(12.50)** |  |  |
| **Medication** |  |  |  |  |  |  |  |  |
| **ACEI, n (%)** | **0(0.00)** | **1(4.17)** |  |  | **0(0.00)** | **2(8.33)** |  |  |
| **ARB, n (%)** | **15(55.56)** | **12(50.00)** |  |  | **21(67.74)** | **14(58.33)** |  |  |
| **Calcium channel blocker, n (%)** | **15(55.56)** | **10(41.67)** |  |  | **16(51.61)** | **11(45.83)** |  |  |
| **Diuretic, n (%)** | **2(7.41)** | **2(8.33)** |  |  | **3(9.68)** | **1(4.17)** |  |  |
| **β‑blocker, n (%)** | **5(18.52)** | **5(20.83)** |  |  | **4(12.90)** | **4(16.67)** |  |  |
| **α‑blocker, n (%)** | **0(0.00)** | **1(4.17)** |  |  | **0(0.00)** | **1(4.17)** |  |  |
| **Centrally acting antihypertensive, n (%)** | **0(0.00)** | **1(4.17)** |  |  | **0(0.00)** | **0(0.00)** |  |  |
| **Untreated, n (%)** | **2(7.41)** | **2(8.33)** |  |  | **1(3.23)** | **0(0.00)** |  |  |
| **24‑hour urine volume, ml** | **2100.00(1500.00,2500.00)** | **1850.00(1425.00,2500.00)** | **-1.050** | **0.294** | **2100.00(1600.00,2500.00)** | **2000.00(1500.00,2500.00)** | **-0.775** | **0.438** |
| **UACR, mg/g** | **687.80(326.70,3347.00)** | **1358.75(385.43,3649.63)** | **-0.698** | **0.485** | **782.40(257.20,4116.80)** | **1024.60(494.73,3534.45)** | **-0.917** | **0.359** |
| **24‑hour urinary protein, mg** | **1875.00(1425.00,2500.00)** | **1558.50(733.75,5391.25)** | **-0.019** | **0.985** | **1875.00(906.00,4296.00)** | **2721.00(1071.75,7242.50)** | **-0.984** | **0.325** |
| **eGFR, mL·min^-1^·1.73 m^-2^** | **78.97±30.20** | **71.42±36.06** | **-0.814** | **0.420** | **81.77±29.02** | **81.37±29.96** | **-0.051** | **0.960** |
| **Scr, µmol/L** | **82.00(68.80,126.00)** | **107.50(73.00,137.50)** | **-1.378** | **0.168** | **84.00(71.00,108.00)** | **87.50(62.50,93.50)** | **-0.577** | **0.564** |
| **CRP, mg/L** | **4.90(3.00,8.40)** | **3.55(2.83,7.23)** | **-1.057** | **0.290** | **3.80(2.50,7.60)** | **3.85(2.48,5.05)** | **-0.221** | **0.825** |
| **Triglycerides, mmol/L** | **1.60(1,11,1.88)** | **1.69(1.00,2.83)** | **-0.557** | **0.578** | **1.70(1.23,2.12)** | **2.20(1.60,3.00)** | **-2.241** | **0.025** |
| **Total cholesterol, mmol/L** | **4.97±1.08** | **5.42±1.81** | **1.077** | **0.287** | **5.26(4.00,5.66)** | **5.29(4.57,6.27)** | **-0.900** | **0.368** |
| **HDL-C, mmol/L** | **1.33(1.21,1.54)** | **1.29(1.03,1.70)** | **-0.944** | **0.345** | **1.33(1.24,1.54)** | **1.24(1.06,1.59)** | **-0.942** | **0.346** |
| **LDL-C, mmol/L** | **2.76±0.73** | **3.08±1.05** | **1.269** | **0.210** | **2.94(2.10,3.42)** | **2.92(2.46,3.64)** | **-0.747** | **0.455** |
| **Platelet count, ×10^9^/L** | **214.00(179.00,237.00)** | **232.50(166.25,247.75)** | **-0.529** | **0.597** | **214.00(189.00,243.00)** | **227.00(181.75,247.00)** | **-0.323** | **0.747** |
| **Alkaline phosphatase, U/L** | **68.70(59.50,85.90)** | **66.75(59.40,81.30)** | **-0.462** | **0.644** | **69.44±18.38** | **71.96±15.01** | **0.545** | **0.588** |
| **Serum albumin, g/L** | **38.64±6.01** | **36.15±9.03** | **-1.143** | **0.260** | **40.30(36.40,43.70)** | **38.25(32.95,41.63)** | **-1.281** | **0.200** |
| **Uric acid, µmol/L** | **320.63±77.09** | **354.13±88.73** | **1.443** | **0.155** | **330.39±73.85** | **375.38±98.68** | **1.935** | **0.058** |
| **Serum phosphorus, mmol/L** | **1.14±0.17** | **1.23±0.18** | **1.807** | **0.077** | **1.16±0.17** | **1.21±0.18** | **0.991** | **0.326** |
| **Serum potassium, mmol/L** | **3.99±0.30** | **4.08±0.50** | **0.846** | **0.402** | **3.99±0.33** | **4.10±0.58** | **0.826** | **0.415** |
| **Serum sodium, mmol/L** | **140.67±2.15** | **139.39±1.99** | **-2.202** | **0.032** | **140.67±2.14** | **139.45±2.07** | **-2.108** | **0.040** |
| **Serum chloride, mmol/L** | **105.57±2.40** | **105.69±2.64** | **0.166** | **0.869** | **105.93±2.57** | **105.27±3.29** | **-0.838** | **0.406** |
| **Serum bicarbonate, mmol/L** | **26.20(24.30,28.50)** | **26.05(23.83,28.80)** | **-0.774** | **0.439** | **26.20(24.30,27.10)** | **25.20(23.25,29.30)** | **-0.772** | **0.440** |

Notes: abbreviations as in Supplementary Table S1.

**Supplementary Table S25.** Hypertension subgroups: Comparison of secondary efficacy outcomes at 3 months among the three groups after PSM

| **Variable** | **Combination**  **(n=27)**  **n** | **Finerenone**  **(n=24)** | **t/Z** | ***P*** | **Combination**  **(n=31)** | **Dapagliflozin**  **(n=24)** | **t/Z** | ***P*** |
| --- | --- | --- | --- | --- | --- | --- | --- | --- |
| **SBP, mmHg** | **121.46±12.00** | **127.56±11.58** | **1.187** | **0.249** | **118.88±9.92** | **121.57±24.47** | **0.386** | **0.704** |
| **DBP, mmHg** | **76.23±14.07** | **80.11±13.87** | **0.640** | **0.530** | **77.00(69.00,81.00)** | **71.00(67.50,80.00)** | **-0.726** | **0.468** |
| **CRP, mg/L** | **3.80(2.28,6.03)** | **2.80(1.70,5.80)** | **-0.791** | **0.429** | **3.45(1.98,6.80)** | **3.30(2.33,4.58)** | **-0.025** | **0.980** |
| **Triglycerides, mmol/L** | **1.52(1.12,2.12)** | **1.76(1.30,2.76)** | **-0.826** | **0.409** | **1.79(1.44,2.51)** | **1.87(1.47,2.68)** | **-0.599** | **0.549** |
| **Total cholesterol, mmol/L** | **5.12±1.08** | **5.38±1.47** | **0.649** | **0.520** | **5.40(4.19,6.18)** | **5.11(3.89,6.15)** | **-0.328** | **0.743** |
| **HDL-C, mmol/L** | **1.54(1.21,1.85)** | **1.28(1.10,1.57)** | **-1.518** | **0.129** | **1.53±0.41** | **1.34±0.41** | **-1.496** | **0.142** |
| **LDL-C, mmol/L** | **2.73±0.63** | **3.02±0.79** | **1.321** | **0.194** | **2.94±0.96** | **2.91±0.97** | **-0.095** | **0.925** |
| **Platelet count, ×10^9^/L** | **207.65±69.34** | **222.29±76.52** | **0.611** | **0.545** | **221.45±83.44** | **218.75±61.59** | **-0.108** | **0.915** |
| **Alkaline phosphatase, U/L** | **77.00(61.00,91.75)** | **58.75(47.98,65.90)** | **-2.516** | **0.012** | **78.22±23.49** | **75.11±21.95** | **-0.449** | **0.656** |
| **Serum albumin, g/L** | **40.37±4.03** | **37.92±7.09** | **-1.366** | **0.182** | **41.70(36.80,43.10)** | **38.70(33.80,43.40)** | **-0.835** | **0.404** |
| **Uric acid, µmol/L** | **335.42±104.06** | **361.00±89.56** | **0.902** | **0.372** | **332.33±102.66** | **375.63±79.11** | **1.671** | **0.101** |
| **Serum phosphorus, mmol/L** | **1.16±0.16** | **1.20±0.16** | **0.883** | **0.382** | **1.17±0.18** | **1.22±0.15** | **1.018** | **0.313** |
| **Serum potassium, mmol/L** | **4.42±0.50** | **4.29±0.57** | **-0.869** | **0.389** | **4.35±0.48** | **4.12±0.43** | **-1.790** | **0.079** |
| **Serum sodium, mmol/L** | **138.86±2.24** | **139.52±1.97** | **1.087** | **0.282** | **139.38±2.17** | **139.18±1.68** | **-0.358** | **0.722** |
| **Serum chloride, mmol/L** | **105.47±2.90** | **104.90±2.78** | **-0.700** | **0.487** | **106.17±2.73** | **105.41±3.45** | **-0.890** | **0.378** |
| **Serum bicarbonate, mmol/L** | **24.94±3.21** | **26.37±2.20** | **1.775** | **0.083** | **25.42±3.38** | **25.34±3.00** | **-0.081** | **0.936** |

Notes: abbreviations as in Supplementary Table S1.

**Supplementary Table S26.** Hypertension subgroups: Comparison of safety at 3 months among the three groups after PSM

| **Adverse reaction** | **Combination**  **(n=27)**  **n** | **Finerenone**  **(n=24)** | ***P*** | **Combination**  **(n=31)** | **Dapagliflozin**  **(n=24)** | ***P*** |
| --- | --- | --- | --- | --- | --- | --- |
| **Incidence of adverse reactions (%)** | **3(11.11)** | **4(16.67)** | **0.693** | **3(9.68)** | **2(8.33)** | **1.000** |
| **Hyperkalemia (%)** | **3(11.11)** | **4(16.67)** |  | **3(9.68)** | **0(0.00)** |  |
| **Urinary tract infection (%)** | **0(0.00)** | **0(0.00)** |  | **0(0.00)** | **1(4.17)** |  |
| **Hypoglycemia (%)** | **0(0.00)** | **0(0.00)** |  | **0(0.00)** | **1(4.17)** |  |

**Supplementary Fig. S1** Cost-effectiveness plane of probabilistic sensitivity analysis (1,000 Monte Carlo simulations). (A) Combination therapy versus Finerenone. (B) Combination therapy versus Dapagliflozin. C, cost; E, effect; ΔC, incremental cost; ΔE, incremental effect; ICER, incremental cost‑effectiveness ratio.
